# A comprehensive analysis framework for evaluating commercial single-cell RNA sequencing technologies

**DOI:** 10.1093/nar/gkae1186

**Published:** 2024-12-16

**Authors:** Marco De Simone, Jonathan Hoover, Julia Lau, Hayley M Bennett, Bing Wu, Cynthia Chen, Hari Menon, Amelia Au-Yeung, Sean Lear, Samir Vaidya, Minyi Shi, Jessica M Lund, Ana Xavier-Magalhães, Yuxin Liang, Ahmet Kurdoglu, William E O’Gorman, Zora Modrusan, Daniel Le, Spyros Darmanis

**Affiliations:** Department of Proteomic and Genomic Technologies, Genentech, South San Francisco, 94080, CA, USA; Department of Proteomic and Genomic Technologies, Genentech, South San Francisco, 94080, CA, USA; Department of Proteomic and Genomic Technologies, Genentech, South San Francisco, 94080, CA, USA; Department of Proteomic and Genomic Technologies, Genentech, South San Francisco, 94080, CA, USA; Department of Proteomic and Genomic Technologies, Genentech, South San Francisco, 94080, CA, USA; Department of Proteomic and Genomic Technologies, Genentech, South San Francisco, 94080, CA, USA; Department of Proteomic and Genomic Technologies, Genentech, South San Francisco, 94080, CA, USA; Department of Proteomic and Genomic Technologies, Genentech, South San Francisco, 94080, CA, USA; Department of Proteomic and Genomic Technologies, Genentech, South San Francisco, 94080, CA, USA; Department of Proteomic and Genomic Technologies, Genentech, South San Francisco, 94080, CA, USA; Department of Proteomic and Genomic Technologies, Genentech, South San Francisco, 94080, CA, USA; Department of Proteomic and Genomic Technologies, Genentech, South San Francisco, 94080, CA, USA; Department of Proteomic and Genomic Technologies, Genentech, South San Francisco, 94080, CA, USA; Department of Proteomic and Genomic Technologies, Genentech, South San Francisco, 94080, CA, USA; Department of Proteomic and Genomic Technologies, Genentech, South San Francisco, 94080, CA, USA; Department of Proteomic and Genomic Technologies, Genentech, South San Francisco, 94080, CA, USA; Department of Proteomic and Genomic Technologies, Genentech, South San Francisco, 94080, CA, USA; Department of Proteomic and Genomic Technologies, Genentech, South San Francisco, 94080, CA, USA; Department of Proteomic and Genomic Technologies, Genentech, South San Francisco, 94080, CA, USA

## Abstract

This study examined nine prominent commercially available single-cell RNA sequencing (scRNA-seq) kits across four technology groups. Each kit was characterized using peripheral blood mononuclear cells (PBMCs) from a single donor, which enabled consistent assessment of factors such as analytical performance, protocol duration and cost. The Chromium Fixed RNA Profiling kit from 10× Genomics, with its probe-based RNA detection method, demonstrated the best overall performance. The Rhapsody WTA kit from Becton Dickinson exhibited a balance between performance and cost. Importantly, we introduce the read utilization metric, which differentiates scRNA-seq kits based on the efficiency of converting sequencing reads into usable counts. Thus, read utilization is an important feature that substantially impacts sensitivity and cost. With data from 169, 262 cells, our work provides a comprehensive comparison of commercial scRNA-seq technologies to facilitate the effective implementation of single-cell studies.

## Graphical Abstract

**Graphical Abstract:**
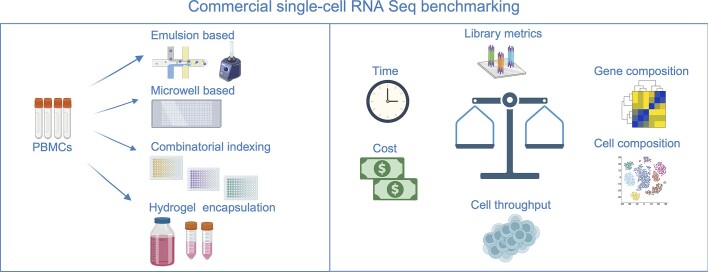

## Introduction

Over the past ten years, the emerging landscape of single-cell technologies has ushered in a new era in biomedical research, unveiling previously uncharted terrains of cellular types, states and lineages (1). Pioneering technologies have increased the number of cells retrieved from a single experiment (2) while continuously reducing the cost per cell. The initial surge in throughput primarily resulted from the development of droplet-based (3,4) and microwell-based methodologies (5,6), which have facilitated the parallel processing of thousands of cells. More recently, the advent of combinatorial-indexing, in which fixed cells serve as reaction chambers for sequential RNA barcoding, has further increased cell throughput. Combinatorial-indexing removes the need to physically isolate each cell, as each cDNA molecule receives a unique combinatorial barcode that distinguishes its cellular origin and enables simultaneous processing of hundreds of thousands of cells (7,8).

The commercialization of scRNA-seq technologies, often originating from academic prototypes, has increased access to these techniques. However, given the diverse range of available technologies, a question arises: How do these different strategies compare in terms of performance and effectiveness? Although several evaluations of the available scRNA-seq approaches exist (9–14), there currently lacks a systematic evaluation of existing and emerging commercial technologies. This knowledge gap hinders our ability to select an optimal scRNA-seq approach tailored to specific investigative needs. By enumerating practical features (e.g. cost and time requirements) and assessing the analytical performance of each technology at the library-level (e.g. read utilization), gene-level (e.g. detection sensitivity) and cell-level (e.g. cell type discrimination); this work is a guide for the optimal selection and effective application of scRNA-seq kits in biomedical research.

**Figure 1.**
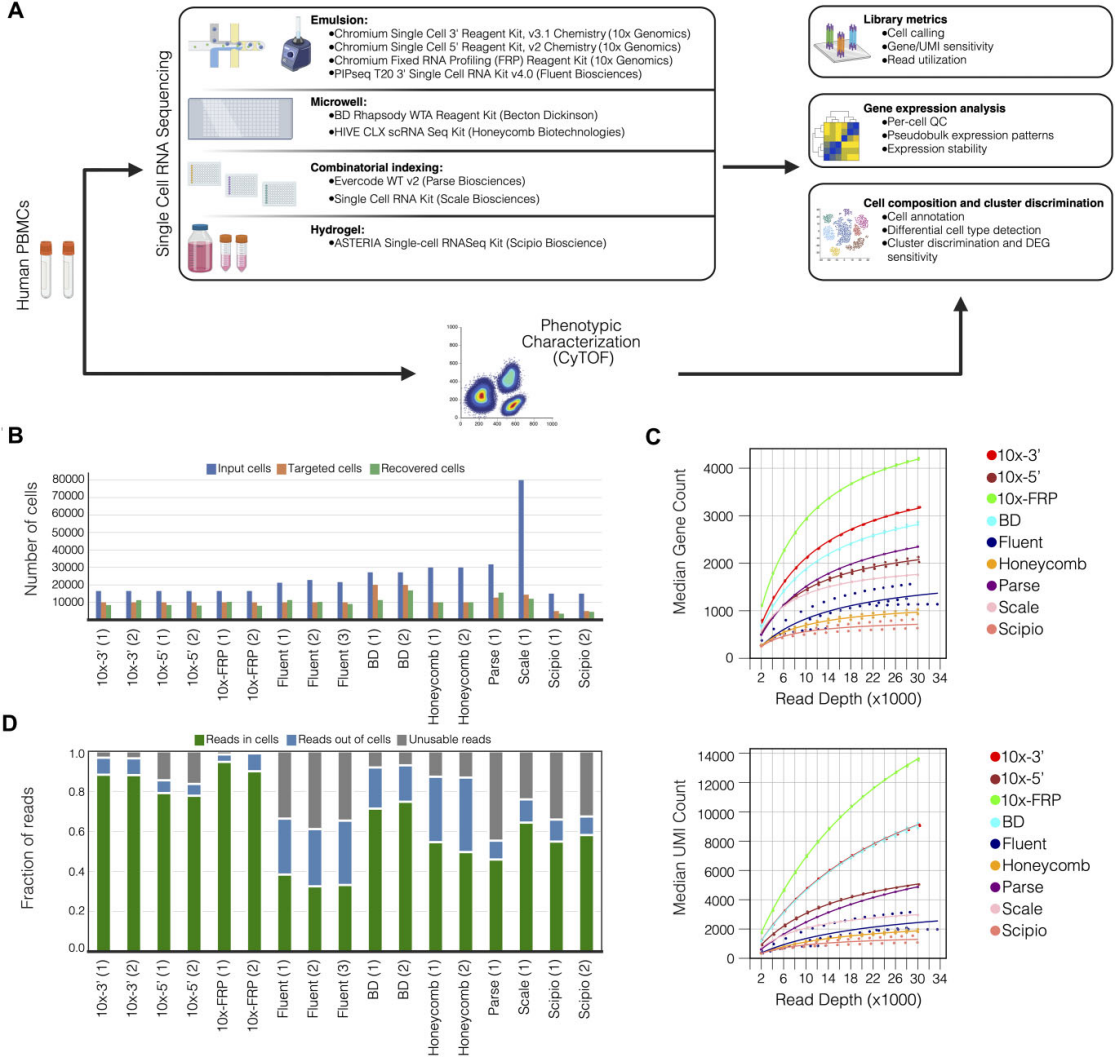
Experimental workflow and library-level metrics. (**A**) Experimental layout. Aliquots of the same PBMC sample were processed using nine different commercially available scRNA-seq kits. The same sample was phenotypically characterized using CyTOF. Following library preparation and sequencing, the protocols were assessed for their performance on the library, gene and cell composition level. Created in BioRender. Darmanis, S. (2024) https://BioRender.com/d29p479 (**B**) Number of input cells (i.e. cells loaded on device) used to yield the desired cell target, number of targeted cells and number of cells recovered by each kit according to the associated cell calling algorithm. (**C**) Number of median genes (top) and UMIs (bottom) detected at downsampled depths from 2000 to 30 000 reads per cell in steps of 2, 000. Downsampled data were aggregated by kit and fitted with a Michaelis-Menten curve to model saturation. (**D**) Read allocation per kit with: the fraction of reads mapped and tagged with a cell barcode, the fraction of reads mapped and tagged with a non-cell barcode and the fraction of unusable reads not mapped (e.g. read duplicates) and/or tagged with a cell barcode. Numbers in parentheses denote replicates for each method.

We categorized the scRNA-seq kits compared in this study into four general technology groups: emulsion-based methods, microwell-based methods, combinatorial-indexing methods and one hydrogel-based method (Figure 1A). To assess performance of the different single-cell RNAseq kits, we used peripheral blood mononuclear cell (PBMCs) from a single donor blood draw. The cell type distribution of this PMBC sample was determined using CyTOF (15). The PBMCs were split into aliquots and used for each kit assessment. For most kits, two independent technical replicates were performed. However, due to initial cell calling variability, we included a third replicate for Fluent. For Parse and Scale, only a single replicate was processed due to high kit costs.

For emulsion-based kits, individual cells are encapsulated in water-in-oil emulsion droplets that are used as reaction chambers. We tested the Chromium Single Cell 3′ reagent kit v3.1 (10 × 3′), the Chromium Single Cell 5′ reagent kit v2 (10 × 5′), the Chromium Fixed RNA Profiling kit by 10x Genomics (10x FRP), and the PIP Seq T20 3′ Single Cell RNA kit v4 by Fluent Biosciences (Fluent). The 10 × 3′, 10 × 5′ and Fluent kits utilize Reverse Transcription (RT) to generate barcoded cDNA molecules from mRNA. As the name implies, the 10 × 5′ kit targets the 5′-end of cDNA for sequencing. Unique among these kits is the 10x FRP kit, which utilizes the oligonucleotide ligation assay (OLA) (16) to measure gene expression in formaldehyde-fixed samples using paired probes. Currently, only mouse or human probe sets are available. Cognate probe pairs were designed to hybridize proximally at target loci, enabling probe ligation and capture in droplets (Extended Data Figure 1a).

With microwell-based kits, cells are separated in microwells that serve as individual reaction chambers for barcoding. We tested the BD Rhapsody WTA reagent kit by Becton Dickinson (BD) and the HIVE CLX scRNA seq v1 kit by Honeycomb Technologies (Honeycomb). Each usable microwell contains a single cell and a magnetic bead conjugated with barcoded oligos. Following cell lysis, mRNA capture and RT yield barcoded cDNA strands that are pooled from all microwells on the device for subsequent pooled second strand synthesis and amplification. BD and Honeycomb use random primers for second strand synthesis (Extended Data Figure 1b).

Combinatorial-indexing methods are based on split-pool indexing of cDNA within fixed cells. Through iterative PCR or ligation, a unique set of barcodes is appended to the collection of cDNA molecules from each cell. We tested two combinatorial-indexing kits: Evercode WT kit v2 by Parse Biosciences (Parse) and the Single Cell RNA kit by Scale Biosciences (Scale). For both kits, cells are fixed and permeabilized, which facilitates intracellular split-pool indexing without the need to isolate individual cells (Extended Data Figure 1c). For Parse, barcodes are added through three rounds of ligation and one round of PCR. The Scale kit follows a similar procedure but only requires two rounds of ligation. Additionally, the Parse kit achieves broad transcript coverage using a mix of barcoded random and oligo-dT primers, whereas the Scale kit covers only the 3′-end of transcripts, since it relies solely on oligo-dT priming.

In the category of hydrogel-based methods, we evaluated the Asteria Single Cell RNA seq kit by Scipio Biosciences (Scipio), which isolates individual cells paired with barcoded capture beads within a hydrogel matrix. After cell lysis, the mRNA content in each cell is hybridized to bead-conjugated oligo-dT sequences containing a unique cell barcode (CB). Then, the hydrogel is dissolved and the captured mRNA undergoes RT and cDNA amplification (Extended Data Figure 1d).

In summary, the rapid advancement and commercialization of scRNA-seq technologies has revolutionized biomedical research, offering unprecedented insights into cellular diversity and function. However, a comprehensive and systematic comparison of current technologies is lacking. This study addresses that knowledge gap by evaluating nine prominent scRNA-seq kits across four major technology groups. By evaluating experimental features and analytical performance metrics at multiple levels, this work provides a valuable resource for selecting an appropriate scRNA-seq approach that best aligns with research aims. This evaluation not only enhances our understanding of the current scRNA-seq technology landscape but also sets the stage for novel applications and innovations in single-cell transcriptomics.

## Materials and methods

### Sample preparation, viability assessment and sequencing

Multiple aliquots of frozen Human PBMCs were purchased from Stem Cell Technologies (Cat No 70025.2). PMBCs were thawed in complete RPMI 1640 Medium (Thermo Fisher Scientific Cat No 11 875 093), washed twice in PBS, and resuspended in PBS 0.04% BSA (Thermo FIsher Scientific Cat No AM2616). Cell concentration and viability were assessed using Cellometer K2 (Nexcelom Bioscience) using dual-fluorescent staining (AO-PI). The viability of all the PBMC aliquots was above 95%. For each kit, PBMC aliquots were processed according to the manufacturer user guide. Barcoded cDNA libraries were sequenced with Illumina sequencers (NextSeq2000 and NovaSeq6000). The sequencing specifications are listed in Table 1 in the *Sequencing Format* column.

**Table 1.** Table of kit descriptions and requirements

Protocols tested	Abbreviation	Technology group	Detection method	Transcipt coverage	Fixation	Sequencing format [R1, R2, I1, I2] (bp)	Required cell loading	Kit cost (USD)	Reactions per kit	Cost per reaction (USD)
Chromium Single Cell 3′ Reagent Kit, v3.1 Chemistry. (10x Genomics)	10 × 3′	Emulsion	Reverse Transcription	3-prime end	no	[28, 90, 10, 10]	16, 000	$26,300	16	$1, 644
Chromium Single Cell 5′ Reagent Kit, v2 Chemistry. (10x Genomics)	10 × 5′	Emulsion	Reverse Transcription	5-prime end	no	[28, 90, 10, 10]	16, 000	$26,300	16	$1, 644
Chromium Fixed RNA Profiling Reagent Kit (10x Genomics)	10X FRP	Emulsion	Probe Hybridization	N/A	yes (CHO)	[28, 90, 10, 10]	16, 000	$6, 900	4	$1, 725
PIPseq T20 3′ Single Cell RNA Kit v4.0 (Fluent Biosciences)	Fluent	Emulsion	Reverse Transcription	3-prime end	no	[54, 67, 8, 8]	40, 000	$3, 600	4	$900
BD Rhapsody WTA Reagent Kit (Becton Dickinson)	BD	Microwell	Reverse Transcription	3-prime end	no	[75, 75, 8, 0]	30, 000	$6, 365	4	$1, 591
HIVE CLX scRNA Seq Kit v1 (Honeycomb Biotechnologies)	Honeycomb	Microwell	Reverse Transcription	uniform	no	[25, 50, 8, 8]	60, 000	$8, 999	8	$1, 125
Evercode WT v2 (Parse Biosciences)	Parse	Combinatorial-indexing	Reverse Transcription	uniform	yes (unknown)	[74, 86, 6, 0]	350, 000	$11,165	1	$11, 165
Single Cell RNA Kit (Scale Biosciences)	Scale	Combinatorial-indexing	Reverse Transcription	3-prime end	yes (MeOH)	[34, 76, 10, 0]	960, 000	$8, 500	1	$8, 500
ASTERIA Single-cell RNASeq Kit (Scipio Bioscience)	Scipio	Hydrogel	Reverse Transcription	3-prime end	no	[28, 75, 6, 0]	15, 000	$4, 240	4	$1, 060

### PBMC phenotypic characterization

The PBMC sample was analyzed using CyTOF with a panel of 37 antibodies (Supplementary Table 1a) (17). PBMC subpopulation frequencies, relative to the number of live cells, were calculated according to the gating strategy in Supplementary Table 1b and Extended Data Figure 2.

### Experimental design

For the majority of evaluated kits, two replicates targeting 10 000 cells were generated using PBMC aliquots derived from a single healthy donor (Only one sample was generated for both Parse and Scale. Fluent had three replicates). Note that Scipio does not allow processing of more than 5000 cells. PBMC aliquots were thawed directly prior to use. All assays were performed according to kit instructions. Sequencing was performed with a target depth of slightly more than 30 000 reads per cell. Downsampling of FASTQ files was needed to yield the targeted 30 000 reads per cell. All downsampling was performed by the software *seqtk* version 1.4 (https://github.com/lh3/seqtk) with the flags sample −2 -s100. Each targeted sub-library was downsampled a single time from the corresponding full-depth FASTQ file.

### Data processing pipelines

Data processing software supplied by each kit vendor was implemented to produce UMI count matrices. For each of the 10× kits, *CellRanger* version 7.1.0 was used. The 10 × 3′ and 5′ kits required the subcommand count and 10x FRP required the subcommand multi. For Fluent, *PIPseeker* version 2.1 was used. For BD, *Rhapsody* version 2.0 was used. For Honeycomb, *BeeNet* version 1.1.3 was used. For Parse, *split-pipe* version 1.1.1 was used. For Scale, *ScaleRna* version 1.3.3 was used. For Scipio, *Cytonaut* version 7 was used. The human reference (Ensembl 98/GENCODE v32) from 10x Genomics (refdata-gex-GRCh38-2020-A) was used for alignment and feature quantification. This reference was adapted for compatibility with all other kit pipelines.

### Cell calling algorithms

Unlike the cell calling algorithms provided with most kits, the software for Fluent and Honeycomb require user input to identify cells. For the Fluent pipeline, a user can select from five outputs with different cell count yields. The outputs are based on five sensitivity levels according to this formula:


\begin{equation*}{{T}_i} = M\ *\ {{10}^{ - \ \left( {0.5 + 0.25{{L}_i}} \right)}},\ i = \left\{ {1,\ 2,\ 3,\ 4,\ 5} \right\}\end{equation*}

where *T* is the UMI threshold for a barcode to be called a cell, *M* is the number of UMIs for the first ranked barcode where the transcript count does not drop by more than 10% of the previous barcode, and *L* is the multiplier for each of the five sensitivities (*i = {1, 2, 3, 4, 5}*). We selected the sensitivity level that yielded cell counts closest to the targeted 10 000 cells. Honeycomb requires users to input the expected cell number and returns exactly this number of barcodes.

### Read utilization analysis

FASTQ reads were classified into three categories: A) uniquely mapped and tagged with a cell barcode, B) uniquely mapped and tagged with a non-cell barcode, and C) all other reads removed or masked by data processing pipelines (e.g. unmapped reads, untagged reads, duplicates). To determine the number of reads in categories A and B, the BAM files outputted from each pipeline were processed with the following command: samtools view -F 256 ‘$bam_file’ | grep ‘$bc_tag’ | sed ‘$sed_pattern’ | sort | uniq -c | sort -nr | awk ‘{print $2 ‘, ’ $1}’; $bam_file: path to BAM file, $bc_tag: cell barcode tag (e.g. CB:Z:), $sed_pattern: pattern to extract full barcode (*e.g*. ‘s/.*CB:Z:\ ([ACGT]*\).*/\1/’). The resultant set of uniquely mapped and barcoded reads were further segregated based on possession of a passing cell barcode (category A) versus a non-cell barcode (category B). The number of reads in category C was determined by subtracting A and B from the number of reads in the input FASTQ file.

### Sensitivity as a function of sequencing depth analysis

Downsampled sub-libraries were generated with total reads equivalent to the target reads per cell, ranging from 30 000 to 2000 reads per cell at steps of 2000. This resulted in 14 sub-libraries per sample, which were processed using respective software accompanying each kit. The output included median number of genes and UMIs per cell at each sampling depth. For each kit, aggregating across replicates, we fit a Michaelis–Menten saturation function (a specific application of the rectangular hyperbolic function) to these data (18). The Michaelis-Menten function is:


\begin{equation*}\nu = frac{{\ {{V}_{max}}\ *\ \left[ S \right]}}{{{{K}_m}\ + \ \left[ S \right]}}\end{equation*}

Here, $\nu$ is the median gene or UMI count per cell, *V_max_* is the saturation level, $[ S ]$ is the read depth, and *K_m_* is the depth that yields half-maximal saturation. Curve fitting was performed using the curve_fit()function from the python package *scipy.optimize* version 1.13.0. Kits were grouped into sensitivity performance tiers based on hierarchical clustering of 34 equidistant expected values along fitted curves derived from each kit.

### Counts matrix preprocessing

Starting with the counts matrix derived from the 30 000 reads per cell library, cells with fewer than 5 genes were removed (19). Doublets were removed using *Scrublet* (20) *version 0.2.3* with a doublet prediction score threshold of 0.25 for all samples. Next, all count matrices were randomly subsampled to a uniform number of cells (after minimum gene number and doublet filters; *n* = 7750). Scipio was excluded from cell subsampling because its maximum yield is 5000 cells. Lastly, genes present in fewer than three cells per sample were removed (19).

### Quality control metrics

Commonly assessed metrics such as gene counts, UMI counts, mitochondrial percentage, and ribosomal protein percentage were identified on count-matched subsamples of cells (*n* = 7750, excluding Scipio; Scipio-rep-1: *n* = 3457, Scipio-rep2: *n* = 4425) passing gene and cell number controls (see *Counts matrix preprocessing*). The *scanpy* package (version 1.9.1) function sc.pp.calculate_qc_metrics() with flags qc_vars=[‘mt’, ‘ribo’] was used to calculate counts and gene metrics. Mitochondrial and ribosomal genes have gene symbols starting with ‘MT-’ or ‘RPS’/‘RPL’, respectively.

### Statistical analysis of kit variables

For per-cell variables such as gene counts, UMI counts, mitochondrial percentage, and ribosomal percentage, non-parametric tests were performed to determine distributional differences among kits. We used the one-way Kruskal–Wallis test (*scipy package*; stats.kruskal()) followed by post-hoc Dunn's tests for pairwise comparisons (*scikit_posthocs* package; posthoc_dunn()). *P* values were adjusted for multiple testing using the Bonferroni procedure.

### Informative feature selection

Highly deviant genes (HDGs) were identified according to this tutorial: https://www.sc-best-practices.org/preprocessing_visualization/feature_selection.html (*scry* package; https://rdrr.io/bioc/scry/src/R/featureSelection.R). HDG determination is based on counts; therefore, they are unaffected by the inconsistent application of normalization procedures (21). To calculate deviance, a python implementation of the deviance calculation was used (see Code availability section).

### Pseudobulk gene expression

Pseudobulk gene expression was computed from UMI counts per million (CPM) across all cells in a given sample. Specifically, pseudobulk expression was defined as mean log-transformed (np.log1p()) CPM. The intersection of expressed genes across all samples was used as the feature set for PCA. The resultant PC loadings (i.e. weights for each gene) were used for GSEA. From the *gseapy* package version 1.1.2, the function prerank (min_size = 50, max_size = 1000, permutation_num = 1000, seed = 6) was used to query PC loadings against the MSigDB C5 collection, GOBP (Gene Ontology, Biological Process) subset: https://www.gsea-msigdb.org/gsea/msigdb/download_file.jsp?filePath=/msigdb/release/2023.2.Hs/c5.go.bp.v2023.2.Hs.symbols.gmt. For correlations between PC embeddings and gene set expression scores, the *Scanpy* function tl.score_genes() was used to compute scores for each pseudobulk sample.

### Dropout rate and gene specificity scores

Drop-out rates and gene specificity scores (22) were computed for each kit using a bootstrap subsample analysis on all cells aggregated by kit (N_boot = 500, N_sample = 7750). Only the intersection of genes detected in all kits was considered. For each bootstrap iteration, subsampled count matrices were CPM normalized prior to gene drop-out and gene specificity computation. Each gene specificity bootstrap iteration was paired to the corresponding drop-out iteration to allow for subsequent comparisons.

For the gene drop-out bootstrap, we calculated drop-out rate as the fraction of cells with CPM values of zero. A drop-out rate of zero indicates that no cells express the gene while a rate of one indicates that all cells express the gene. The drop-out rate was modeled as a function of pseudobulk gene expression (mean expression per gene across all cells; CPM) using the following exponential decay equation (9):


\begin{equation*}DropOut = a\ \star \ {{e}^{ - b\ \star \ x}}^{}\end{equation*}

Where $x$ is the pseudobulk gene expression (CPM) for a given gene, $b$ is the decay rate parameter and $a$ is the scale parameter that defines the max drop-out value. The scale parameter $a$ was set to a value of 1 to reflect complete dropout at zero pseudobulk expression. Curve fitting was performed in each bootstrap iteration using the curve_fit()function from the python package *scipy.optimize* version 1.13.0. To optimize curve fitting, initial value estimate for $b$ was randomly initialized to a value between 0 and 0.05. The $b$ parameter was bounded between 0 and 0.05. Estimated parameters across all bootstraps were used to compute bootstrapped mean estimates and 95% confidence intervals. Half-maximal gene drop-out values (GD50) for each bootstrap iteration were computed as follows:


\begin{equation*}GD50 = - \ \frac{{ln\ \left( {0.5} \right)}}{b}\end{equation*}

For the gene specificity bootstrap, specificity was computed according to the methodology by Martinez and Reyes–Valdes (22) using a python implementation of the entropySpecificity() function in the *BioQC R* package (23). Gene specificity for a given gene $i$ is an extension of Shannon entropy and is defined as:


\begin{equation*}{{S}_i} = \frac{1}{t}\ \left( {\mathop \sum \limits_{j = 1}^t \frac{{{{p}_{ij}}}}{{{{p}_i}}} \star \ lo{{g}_2}\ \left( {\frac{{{{p}_{ij}}}}{{{{p}_i}}}} \right)} \right)\end{equation*}

Where ${{S}_i}$ is the gene specificity score for a given gene, $t$ is the number of cells in a sample (7750 for this bootstrap), ${{p}_{ij}}$ is the proportion of CPM from gene $i$ in cell $j$. The following equation defines the average CPM proportion of gene $i$ across all cells, ${{p}_i}$:


\begin{equation*}{{p}_i} = \frac{1}{t}\ \mathop \sum \limits_{j = 1}^t {{p}_{ij}}\end{equation*}

For each kit, gene specificity score densities across all bootstrap iterations were plotted using sns.kdeplot() from *seaborn* and stats.gaussian_kde() from *scipy* was used to find the distribution peak.

### Integration of cells across kits

The *Harmony* (*harmonypy version 0.0.9*) integration algorithm was used to generate adjusted PCA embeddings that account for kit-specific variation (21,24). The top 2, 000 batch-corrected HDGs across all samples were selected as the feature set. Specifically, deviance was computed per sample (batch) and the median deviance across all samples was used to rank genes. Next, normalization and scaling were performed independently for each sample (batch-specific processing) using the following *Scanpy* version 1.9.1 functions with default settings: 1) pp.normalize_total(), 2) pp.log1p() and 3) pp.scale(). The integration step was performed using the *Scanpy* wrapper function (*scanpy.external*) pp.harmony_integrate(); key = ‘$kit_id’ (*i.e. Anndata* object .obs column name for kit identities), basis = ‘X_pca’, adjusted_basis = ‘X_pca_harmony’). The resulting adjusted PCA embeddings were used to construct a neighborhood graph for UMAP visualization of all integrated cells: sc.pp.neighbors(); n_neighbors = 100, n_pcs = 15.

### Cell annotation

The *SeuratData R* package version 0.22 was used to access a dataset of annotated PBMCs derived from several scRNA-seq methods (12) (loaded using data (‘pbmcsca’); data version: 3.0.0). This reference set consists of ten cell type labels: B cells, CD14 + monocytes, CD16 + monocytes, CD4 + T cells, CD8 + T cells (called Cytotoxic T cells in the dataset), Natural killer cells, Plasmacytoid dendritic cells, Dendritic cells, Megakaryocytes and Unassigned cells. The reference cells were split into training and validation sets. The validation set was created by random subsampling to yield 7750 cells to match the number of cells in each kit. The training set consisted of 20 840 cells. These data sets were used to train reference-to-query label transfer models.

Cells were annotated using the consensus of two independent label transfer methods, CellTypist (25) and Seurat's Label Transfer (26). Cells with discordant labels across the two methods were labeled ‘Unassigned’. Next, the cells from all kits were integrated using the *Harmony* algorithm. The cells segregated into two coarse groups consisting of mostly lymphocytes and monocytes. For each coarse group, Leiden overclustering (i.e. clusters >> labels; *n* ∼ 20) was performed to yield high-granularity subpopulations primarily composed of a single dominant cell type label. The majority label within each subcluster was used to relabel all constituent cells. The following provides detailed methodology:

#### *CellTypist* version 1.6.2.

The implementation of *CellTypist* was based on this tutorial: https://colab.research.google.com/github/Teichlab/celltypist/blob/main/docs/notebook/celltypist_tutorial_cv.ipynb. Each query (*i.e*. an individual sample dataset) was processed independently as follows: 1) 2000 HDGs from the query were identified, 2) UMI counts per cell were normalized to 10 000 (*scanpy* package; pp.normalize_per_cell(); counts_per_cell_after = 10 000), and 3) counts were log1p transformed. Scaling was performed by *CellTypist*. Batch-normalization (i.e. across scRNA-seq methods) of the reference set was performed similarly to above. During the *CellTypist* model training on the reference set, approximately 1300 (i.e. intersection of top 300 explanatory variables for each label) of the original 2000 HDGs were selected as the final feature set. Label transfer we performed using the argument majority_vote = True.

#### *Seurat label transfer* version 4.1.1 (*R* version 4.2.0)

Label transfer was based on this *Seurat* tutorial: https://satijalab.org/seurat/articles/integration_mapping.html. The reference dataset was processed as follows: 1) data was partitioned by method, 2) each reference method was independently normalized (NormalizeData; default settings) 3) and the top 2000 variable genes were identified (FindVariableFeatures; selection.method = ‘vst’, nfeatures = 2000). The first 30 principal components were used to find anchors for reference data integration (FindIntegrationAnchors; dims = 1:30, scale = TRUE, reduction = ‘cca’). For each query dataset, raw counts were normalized and the first 30 principal components were used for label transfer (FindTransferAnchors; dims = 1:30, reference.reduction = ‘pca’ and TransferData; dims = 1:30). *SeuratDisk* version 0.0.0.9020 was used to interconvert between *Seurat* and *Scanpy* objects.

#### Leiden overclustering and reclassification

After consensus annotation, an overclustering approach we implemented similar to the majority voting method employed by *CellTypist*: 1) The integrated cell population was divided into two coarse-grain groups composed of mostly lymphocytes and monocytes (sc.tl.leiden(); res = 0.002), 2) Each group was independently reprocessed to yield normalized and scaled data, and 3) For each of these groups, the Leiden algorithm was used to over-cluster cells into at least 20 clusters. To determine the resolution needed for at least 20 clusters, a 30 000 cell subsample was used to more efficiently scan across a range of resolutions. The initial resolution was 1.25 and incremented in steps of 0.05. The resolution values of 1.65 and 1.35 were found to be optimal resolutions for the lymphocyte and monocyte subsamples, respectively. These resolutions were used to initiate a scan on the full dataset, which found resolutions 1.8 and 1.4 to yield at least 20 clusters for the full lymphocyte and monocyte groups, respectively. All cells within a fine-grained cluster were subject to reclassification if two conditions were satisfied: 1) the majority (at least 60%) of cells shared the same consensus label and 2) the majority cell type was not ‘Unassigned’. Otherwise, the original consensus labels remained unchanged.

### Cell type distribution analysis

For each kit, bootstrapped cell type proportions were calculated by resampling (with replacement) aggregated replicates. Resampling was performed 500 times to yield subsamples consisting of 7882 cells (equivalent to the number of cells found in Scipio, the sparsest replicated dataset). Mean cell type proportions were calculated using the 500 subsamples. With respect to mean cell type proportions, hierarchical clustering (*scipy* package; cluster.hierarchy.linkage(); method=‘ward’) was used to compare kits in relation to CyTOF. Megakaryocytes and Unassigned cells were excluded since these labels were not included in the CyTOF annotation. In addition, using the 500 subsamples for each kit, mean absolute difference relative to CyTOF cell type proportions were calculated.

### Cell partitioning scores

Kit replicates were concatenated using respective filtered count matrices (*anndata* package; ad.concat()). For each kit, the intersection of deviant genes across replicates were used as features in PCA (*scanpy* package; tl.pca(); svd_solver=‘arpack’, use_highly_variable = True). Concatenated datasets were integrated using Harmony (*scanpy external* package; pp.harmony_integrate(); max_iter_harmony = 30). Then, each dataset was subsampled to the number of cells found in Scipio, the sparsest multiple replicate dataset (*scanpy* package; pp.subsample(), *n* = 7882). This was followed by construction of a neighborhood graph (*scanpy* package; pp.neighbors(); n_neighbors = 30, n_pcs = 15). Leiden clustering was performed over a series of resolutions ranging from 0.02 to 1.00 in increments of 0.02 (*scib* package; metrics.cluster_optimal_resolution()). This process identified the optimum lowest resolution that yielded the smallest difference between the expected number of clusters and labels. All subsequent grouping-based scores were derived from clustering results at the optimum resolution of each kit. Each metric was performed using the following set of tools: ARI (*sklearn* package; adjusted_rand_score()), AMI (*sklearn* package; adjusted_mutual_info_score()), ASW (*scib* package; metrics.silhouette()), bARI (*balanced_clustering* package; balanced_adjusted_rand_index()), bAMI (*balanced_clustering* package; balanced_adjusted_mutual_info()) and CVR was computed as the sum of the first 10 PCA variance ratios.

### Differential gene expression (DGE) analysis

Using the filtered count matrix with cell type annotations, each sample was processed as follows: 1) The number of cells was subsampled to *n* = 3457 (i.e. the lowest cell yield; Scipio replicate 1), 2) Any cell type with fewer than 10 cells was removed from subsequent DEG analysis and 3) A two-sided Wilcoxon rank-sum test was performed for each query cell type versus the remaining cell types (*scanpy* package; tl.rank_genes_groups(); method = ‘wilcoxon’). The set of tested genes includes both highly deviant (those used for PCA, clustering, and embedding) and non-highly-deviant genes with detected counts from each kit. Annotation of significant differential expression required log2 fold-change > 2 and Benjimini–Hochberg-adjusted *P* value < 0.05. For DEG counts aggregated by kit rather than individual samples, mean DEG counts were provided. For each cell type, Z-score normalization was performed using the DEG counts distribution across kits (*scipy* package; stats.zscore()). Hierarchical clustering of kits with respect to cell-type specific DEG count Z-scores was performed using the *seaborn* package (clustermap(); standard_scale = None)

### Tiering system

For cell recovery tiers, observed yields within 20% of the target number of cells were classified as *Tier 1*. All other kits are in *Tier 2*. For cost per cell, equipment cost, and read utilization a kernel density estimator (*scipy* package; stats.gaussian_kde()) was used to identify natural breaks (*scipy package;*signal.find_peaks()), since the data is a one dimensional vector. For equipment cost, a natural log transform was applied (log1p). For maximum cell recovery the data were divided into three tiers to reflect cell counts in typical experimental ranges: *Tier 1)* >20 000 cells, B) 10 000–20 000 cells and *Tier 3)* <10 000 cells. For protocol time, the data were divided into three tiers to reflect the number of work days required to complete each experiment: *Tier 1)* approximately 1 work day (<8.5 h), *Tier 2)* approximately 2 work days (8.5–16 h), and *Tier 3)* more than 2 days (>16 h). For mitochondrial fraction, the data were divided into three tiers to reflect typical ranges considered in quality control analysis: *Tier 1)* Approximately 0%, *Tier 2)* <10%, and *Tier 3)* >10%. For ribosomal protein fraction, the data were divided into two tiers to reflect the bimodal nature of the ribosomal fraction we detected: *Tier 1)* Approximately 0% and *Tier 2) >*0% (most were around 10%). For the remaining performance categories, hierarchical clustering was used to identify performance groupings, since the data for each category were multi-dimensional. For genes and UMIs per cell, hierarchical clustering was performed using both median genes and UMIs per cell.

### Normalized feature score for visualization

The following metric values from Figure 4B were used for visualization: Protocol Time, Cost (Equipment), Cost (per Cell), Max Cell Yield, Cell Yield (% of Target), Read Utilization, Gene Saturation, UMI Saturation, Genes (per Cell), UMIs (per Cell), Gene Expression Stability, CyTOF Similarity, Differential (Gene) Expression, Cluster Discrimination and Mito QC. Normalized feature scores were designed to range from 0 (non-optimal) to 1 (optimal). To generate these scores, the vector of metric values across kits was min-max normalized. In cases where the optimal outcome is inversely proportional to the metric value, 1 minus the min-max-normalized metric values is reported. These categories include Protocol Time, Cost (Equipment), Cost (per Cell), Mito QC, CyTOF Similarity (cophenetic distance score), and Gene Expression Stability (Gene Dropout GD50). Circle size indicates normalized feature score (small = non-optimal; large = optimal). Hierarchical clustering of the normalized feature scores was performed using the *seaborn* package (clustermap(); standard_scale = None).

### Metagene read coverage

Using *pysam* version 0.22.0, CB-tagged BAM files derived from each data processing pipeline were filtered for reads containing barcodes from valid cells. The resultant filtered BAM files were used to generate read coverage BigWig (.bw) files via the subcommand bamCoverage from *deeptools* version 3.5.0. Next, the .bw files were processed using subcommands computeMatrix (–skipZeros –metagene) and plotProfile to visualize average read coverage profiles across genes.

### Illustrations

Figure 1A was created in BioRender. Darmanis, S. (2024) https://BioRender.com/d29p479. Extended Data Figure 1A was created in BioRender. De Simone, M. (2024) https://BioRender.com/h31d907. Extended Data Figure 1B was created in BioRender. De Simone, M. (2024) https://BioRender.com/b77q103. Extended Data Figure 1C was created in BioRender. De Simone, M. (2024) https://BioRender.com/q95t942. The graphical abstract was created in BioRender. De Simone, M. (2024) https://BioRender.com/p10v259. General graphics manipulations were performed using Adobe Illustrator.

### Use of Large Language Models (LLMs)

An enterprise implementation of Chat-GPT was used for proofreading sections of the manuscript and for generating data visualization code. All LLM outputs were reviewed for accuracy.

## Results

### Library metrics

For each evaluated kit, PBMC aliquots were processed according to the user manual. Generally, each device was loaded such that the expected recovery was 10 000 cells (Table 1). Due to the maximum cell yield for the Scipio kit, only 5000 cells were targeted. For the BD kit, preliminary unpublished results indicated cell recovery around 50% of projected yield; therefore, we targeted 20 000 cells with the expectation of recovering the desired 10 000 cells. The number of recovered cells was determined using companion cell calling algorithms provided with each kit's software (Figure 1B). Notably, the cell calling algorithms for both Honeycomb and Fluent require user-defined parameter selection that substantially impacts observed recovery. Mean cell recovery based on the number of targeted cells ranged from approximately 70% (BD) to 122% (Parse) (Supplementary Table 2). Consistent with aforementioned preliminary results, BD demonstrated the lowest cell recovery, with one replicate yielding 43% fewer cells than expected according to loading recommendations. To investigate the effect of sequencing depth on cell recovery, we downsampled individual data sets to yield sub-libraries spanning 2000 to 30 000 reads per cell. Surprisingly, Fluent exhibited substantially higher cell recovery variation compared to other kits (Extended Data Figure 3a,b), likely due to its unique cell calling algorithm which defines the valid cell threshold based on UMI count stability rather than the typical UMI count inflection point (see ‘Materials and methods’). Additionally, Honeycomb exhibited 100% cell recovery with no variation between replicates, according to its accompanying cell calling software. The Honeycomb cell caller returns the exact number of targeted cells, which is a user-defined parameter provided to the software.

Using the downsampled sub-libraries aggregated by kit, we generated total gene and UMI counts at each sequencing depth (Figure 1C). These data were fitted using the Michaelis-Menten saturation model to estimate the maximum number of identifiable genes and UMIs and the number of reads required to reach half-maximal saturation. Hierarchical clustering of the fitted curves indicates three performance tiers based on median detected genes: *Tier 1)* 10× FRP, *Tier 2)* 10 × 3′, BD, 10 × 5′, Parse, and Scale, and *Tier 3)* Fluent, Honeycomb, and Scipio (Extended Data Figure 3c,d). For median recovered UMIs, we observed two performance tiers with 10× FRP, 10 × 3′, and BD performing better than the remaining kits. For both gene and UMI counts, 10× FRP followed by 10 × 3′ and BD exhibited the steepest saturation curves and reached the highest number of attainable genes and UMIs (Supplementary Table 3).

Using the 30 000 reads per cell sub-library, we analyzed the proportion of reads that were mapped, CB-tagged and counted toward gene expression (Figure 1D). The mean proportion of usable reads that were both mapped and associated with a called cell ranged from 34.8% (Fluent) to 92.4% (10× FRP) (Supplementary Table 4). Next, we calculated the average UMI recovery (total UMI counts / total fastq reads) and identified three tiers: *Tier 1)* 10× FRP, *Tier 2)* 10 × 3′ and BD and *Tier 3)* 10 × 5′, Parse, Scale, Fluent, Honeycomb and Scipio (Extended Data Figure 4a,b,c). These tiers differentiate kits into groupings based on the relative utilization of reads, with mean fractions ranging from 0.05 (Scipio) to 0.44 (10× FRP), which ultimately contribute to gene expression counts (Supplementary Table 5)

### Gene composition

**Figure 2.**
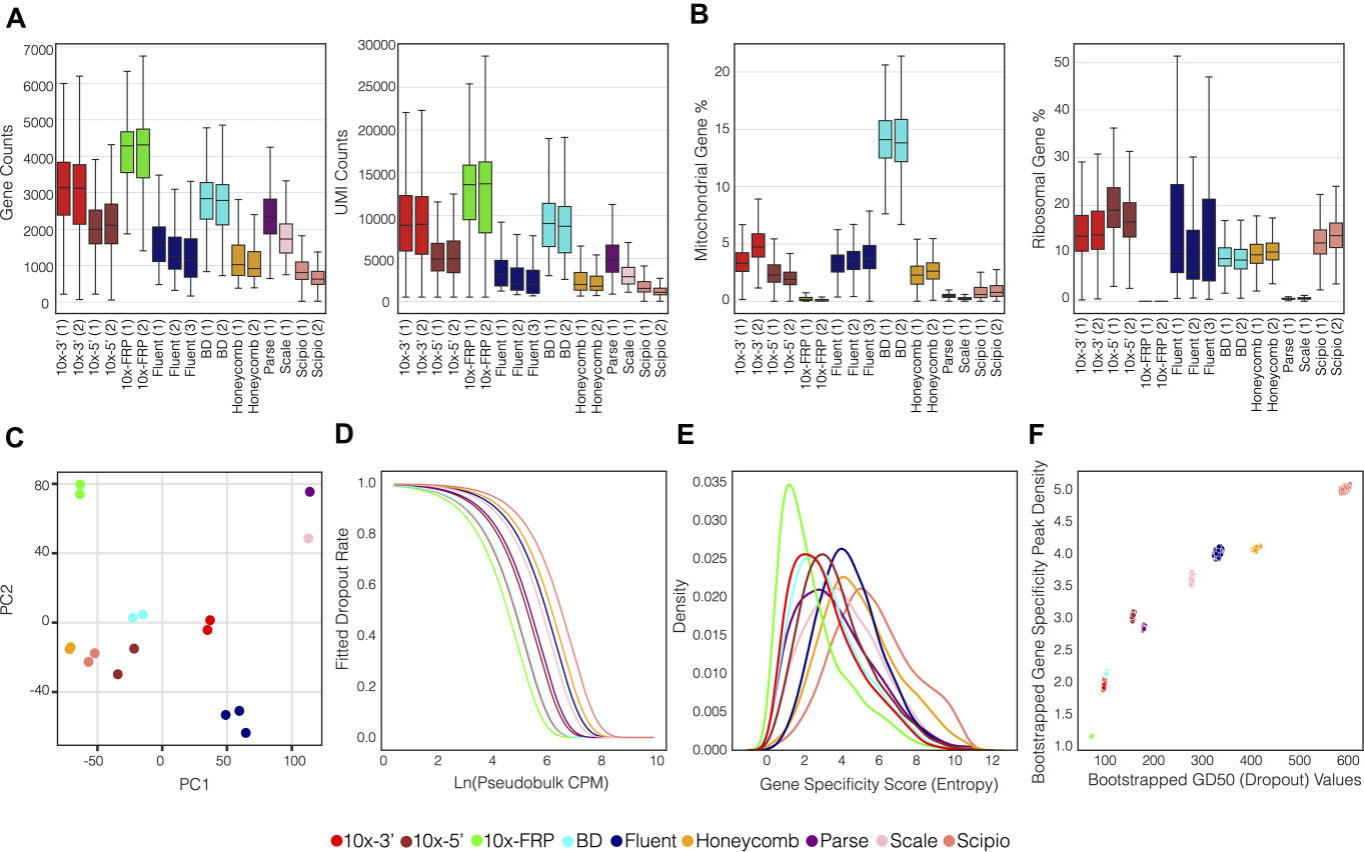
Gene-level metrics. (**A**) The distribution of genes (left) and UMIs (right) recovered for each kit. (**B**) The distribution of MT gene (left) and RP gene (right) percentages for each kit. (**C**) PCA embeddings based on pseudobulk mean gene expression. Numbers in parentheses denote replicates for each method. (**D**) Exponential decay fitted values of mean bootstrapped dropout rate as a function of pseudobulk expression (CPM) colored by kit. (**E**) Density curves of mean bootstrapped gene specificity scores colored by kit. (**F**) Scatter plot of half-maximal gene drop-out (GD50) rates versus peak gene specificity scores across 500 paired bootstrap iterations colored by kit.

Next, we examined the number of detected genes and UMIs per cell derived from each kit using count-matched subsamples of cells (*n* = 7750, excluding Scipio; Scipio-rep-1: *n* = 3457, Scipio-rep2: *n* = 4425) passing quality controls for both metrics (Figure 2A; Supplementary Table 6). Hierarchical clustering of median gene and UMI counts per cell revealed two tiers with 10× FRP, 10 × 3′ and BD yielding significantly higher gene and UMI counts than the remaining kits (Kruskal–Wallis P value < 0.01, post-hoc Dunn's test, Bonferroni-adjusted *P* value < 0.01) (Extended Data Figure 5a; Supplementary Tables 7-Supplementary Tables 8). Tier 1 kits exhibited median gene counts per cell ranging from 2793 to 4312, while Tier 2 kits displayed a substantially lower range of 627–2324. Likewise, Tier 1 median UMI counts per cell ranged from 8779 to 13 719, which was markedly higher than the 1063–4994 range demonstrated by the Tier 2 kits.

Mitochondrial (MT) percentage is a feature often considered in scRNAseq analyses because it can both inform biological function (27,28) and serve as an indicator of cellular stress (29–32). Across the evaluated kits, we observed significant differences in MT percentage per cell (Kruskal–Wallis *P* value < 0.01) (Figure 2B, left panel). In particular, BD displayed a substantially higher MT percentage compared to other kits, which is in agreement with a recently published evaluation (33). This may be due to unique lysis conditions that disrupt both the cell and MT membranes or lower cell viability after loading on device. Conversely, the 10× FRP, Parse, and Scale kits showed relatively low MT percentages (post-hoc Dunn's test, Bonferroni-adjusted P value < 0.01) (Supplementary Tables 9–10). Notably, 10× FRP targets only 11 out of 13 MT genes (Supplementary Table 11). However, even with a comparison of MT percentage using the 11 MT genes detected in all kits, the 10× FRP kit still demonstrated reduced MT percentage. (Extended Data Figure 5b). The remaining variation in MT percentage may be due to kit-specific experimental attributes such as cell processing and lysis buffer. Ribosomal protein (RP) gene percentage is another important feature because RP transcripts typically account for a large portion of sequencing reads (34) and differential RP expression can distinguish cellular function (35). Most kits exhibited RP percentages around 10% of overall transcripts, while 10x FRP, Parse, and Scale exhibited significantly lower RP content (Kruskal–Wallis *P* value < 0.01; post-hoc Dunn's test, Bonferroni-adjusted *P* value < 0.01) (Figure 2B, right panel; Supplementary Tables 9, Supplementary Tables 12). For the 10× FRP kit, this observation is consistent with the fact that it does not contain probes against RP transcripts. The relatively low RP fraction exhibited by Parse has also been reported in a separate comparative analysis (10).

The 10× FRP, Parse and Scale kits are unique among the evaluated approaches because they exhibited relatively low MT and RP percentages in addition to being the only protocols that require cell fixation and permeabilization. Previous studies that investigated the effect of cell fixation and permeabilization on scRNA-seq performance show a similar reduction in RP and MT fractions for fixed cells relative to fresh cells (36–38). These studies posit that fixation and permeabilization can lead to loss of transcripts from the cytosolic compartment. Because the cytoplasm harbors the majority of both RP and MT mRNAs, a corollary of this process would be the relative overrepresentation of nuclear transcripts in fixed cells. Several studies have suggested that a distinguishing feature of nuclear transcripts is a relatively high degree of intron retention due to incomplete splicing (12,39,40). Therefore, the observation that both Parse and Scale exhibit relatively high intronic versus exonic read alignments is consistent with the expected over-representation of nuclear transcripts due to fixation and permeabilization (Extended Data Figure 5c). While the evidence is incomplete in the case of 10× FRP, which lacks intron-targeting probes, these results suggest that the observed reduction in both RP and MT read fraction may also be due to the fixation and permeabilization process.

Next, we evaluated the relative gene expression across kits at the pseudobulk-level. There were 11, 186 genes detected with all kits, while the number of genes private to individual kits ranged from 1 (Scipio) to approximately 350 (Fluent and Scale) (Supplementary Table 13a). Focusing on the top-25 highly expressed genes among the kits, we observe that the combinatorial-indexing kits (Parse and Scale) exhibit the highest fraction of unique highly expressed genes (13/30 or 43%; Parse: 7, Scale: 6), with an additional 9 genes shared between the pair (Extended Data Figure 5d; Supplementary Table 13b). Moreover, considering only commonly detected genes, pseudobulk quantitative expression (mean UMI counts per million) profiles were calculated for each sample. We observed excellent correlation among replicates (median Spearman *r* = 0.99) and comparisons between kits exhibited good agreement (median Spearman *r* = 0.85) (Extended Data Figure 5e). The combinatorial-indexing kits generally displayed lower correlations with other methods (median Spearman rho = 0.75), particularly versus Honeycomb and Scipio (Spearman r ∼ 0.62). To identify distinguishing gene expression factors, we performed principal component analysis (PCA) followed by hierarchical clustering of the first two principal component (PC) embeddings. This revealed that both the combinatorial-indexing kits and 10x FRP are located at the extremes of each axis, apart from the remaining kits (Figure 2C; Extended Data Figure 5f).

Using the PC loadings, we investigated the impact of gene length, gene GC content and total UMI counts on expression. Strikingly, PC1 loadings exhibited a strong positive correlation with gene length (Pearson *r* = 0.68), which partly determines the distribution of kit embeddings along the PC1 axis (Supplementary Table 14). In particular, 10× FRP and the combinatorial-indexing kits are on opposite extremes of the PC1 axis, indicating that gene length can explain a portion of the differential expression between these groups. Conversely, we observed a negative correlation between PC1 and GC content (Pearson r = -0.41). However, there is substantial collinearity between gene length and GC content (Pearson *r* = -0.57), so the relative influence of these factors is hard to determine (Extended Data Figure 5g). Because total UMI counts impact scRNA-seq expression quantitation, we investigated the correlation between PC loadings and pseudobulk total UMI counts per kit. This revealed a strong positive correlation between total UMI counts and PC2 (Pearson *r* = 0.60), suggesting that library complexity has a significant effect on expression analysis.

For PC2, we noticed that protocols requiring fixation and permeabilization (10x FRP, Parse, and Scale) aggregate at the positive end of the axis, consistent with a moderate negative correlation between MT expression and PC2 embeddings (Pearson *r* = -0.42) and a much weaker correlation to PC1 embeddings (Pearson *r* = 0.02) (Supplementary Table 14). Gene set enrichment analysis (GSEA) using PC loadings queried against Biological Process (BP) gene sets from the Gene Ontology (GO) collection corroborates the inverse proportionality between MT content and PC2 loadings. Gene sets associated with mitochondrial functions like ‘proton transmembrane transport’, ‘electron transport chain’ and ‘cellular respiration’ were among the significant negatively enriched terms (Extended Data Figure 5h). Overall, these results suggest that technical factors can have differential impacts across kits with respect to both global and pathway-specific gene expression.

Next, we investigated the stability of gene expression among the kits, specifically focusing on the drop-out rate of each gene. The drop-out rate was computed using bootstrapped cell populations, sampling from aggregated replicates of each kit. The observed exponential decay of drop-out rate relative to psuedobulk expression indicates that the proportion of non-zero expressing cells increases with higher aggregate expression level (Figure 2D). Because drop-out rate only captures the binary variation between expressing versus non-expressing cells, we also computed the gene specificity score (an extension of Shannon entropy) (22), which evaluates gene expression as a continuous variable. Thus, going beyond drop-out rate, gene specificity is able to also capture the variation in gene expression level. Using the aforementioned bootstrapped cell populations, gene specificity distributions were computed for each kit to evaluate the variability of expression within and among cells (Figure 2E). We observed that pseudobulk expression at half-maximal gene drop-out rate (GD50) non-linearly and positively correlates with gene specificity distribution peaks. Notably, the bootstrapped sample sets of each kit were discretely localized (Figure 2F; Supplementary Table 15). Hierarchical clustering of these values identified three tiers: *Tier 1)* 10× FRP, BD, 10 × 3′, 10 × 5′, and Parse *Tier 2)* Fluent, Honeycomb, Scale, and *Tier 3)* Scipio (Extended Data Figure 5i). The Tier 1 kits demonstrated the highest gene expression stability, which translates to improved variance estimates that impact downstream statistical tests, such as those used for differential gene expression analysis.

### Cell composition

**Figure 3.**
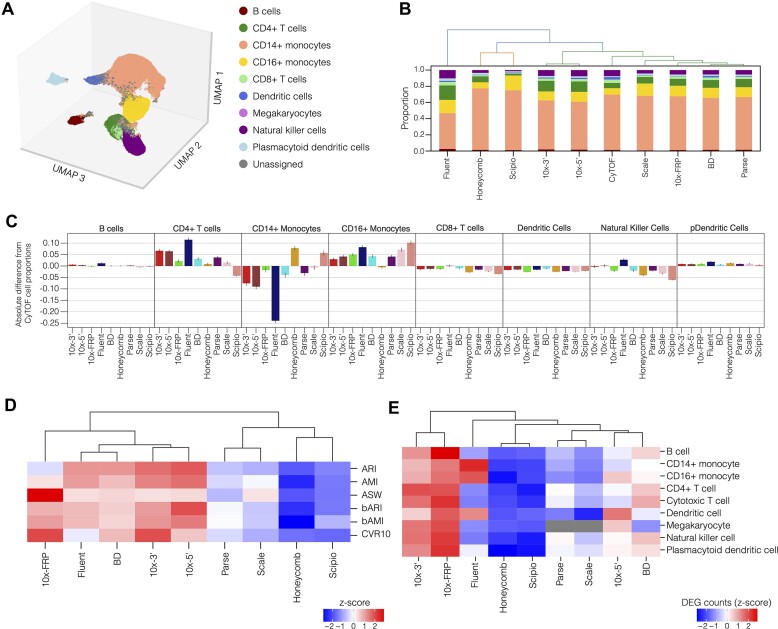
Cell type distribution, partitioning, and differential gene expression analysis. (**A**) Three-dimensional uniform manifold approximation and projection (UMAP) of integrated data, colored by annotated cell type. Unassigned cells are those with discordant labels between Seurat and CellTypist that could not be resolved with Leiden cluster smoothing (**B**) Mean bootstrapped cell type distribution ordered by hierarchical clustering dendrogram. Cell types are colored according to the legend in Figure 3A. (**C**) Mean bootstrapped absolute difference from CyTOF reference cell proportions for each cell type and method. Error bars represent the 95% bootstrapped confidence interval. (**D**) z-score of cell partitioning metrics, organized by hierarchical clustering dendrogram. Metrics included: bAMI, bARI, AMI, ARI, ASW and CVR. (**E**) Mean DEG count z-scores per cell type across, ordered by hierarchical clustering dendrogram.

To systematically annotate cell types across different kits (Figure 3A), we first labeled cells using the agreement of two independent reference-to-query label transfer methods, CellTypist (25) and Seurat Label Transfer (26), trained on an annotated PBMC dataset derived from various scRNA-seq technologies containing ten cell type labels (12) (Extended Data Figure 6a–c). The label agreement procedure demonstrated 94% precision, 81% recall, 86% F1 score (all weighted by class size) and an overall accuracy of 81% on a validation set (Supplementary Table 16). We then refined the initial cell labels using Leiden clustering and local smoothing of integrated cells across all of the kits (Extended Data Figure 6d–j). This approach leverages similar expression patterns exhibited by cells within Leiden clusters to recover unassigned cells (discordant labels between Seurat and CellTypist) and to update cell labels according to neighborhood affiliation, yielding 96.2% cell annotation across all kits (Extended Data Figure 6k).

We aimed to assess the fidelity of cell type proportions determined by each kit relative to those obtained with CyTOF. With accuracy and specificity comparable to standard flow cytometry (15), CyTOF was used to ascertain the reference distribution of cell types based on 40 markers specific to PBMCs. Hierarchical clustering of bootstrapped cell type proportions derived from each kit revealed that Fluent, Honeycomb and Scipio exhibit large deviations from the CyTOF reference distribution, especially for CD14 + monocytes (Figure 3B,C; Supplementary Table 17). Strikingly, Fluent exhibited the largest magnitude deviations in absolute proportion relative to CyTOF: nearly 25% fewer CD14 + monocytes and greater than 10% more CD4 + T cells. It is tempting to speculate that the unique vortexing-based cell-encapsulation methodology employed by Fluent impacts cell type recovery. For instance, this method may bias against delicate cells that are potentially damaged by agitation while enriching for more robust cell types. More generally, differences in sample preparation along with kit-specific permeabilization and lysis reagents are likely to influence cell type recovery.

### Cluster discrimination

For each scRNA-seq kit, we sought to assess the partitioning of cell types into discrete, homogenous groups. Toward this end, data generated using each kit were individually processed to identify cell embeddings based on kit-specific transcriptomic features. Using these embeddings, we assessed various performance metrics for distinguishing cell type groups: balanced Adjusted Mutual Information (bAMI), balanced Adjusted Rand Index (bARI) (41), Adjusted Mutual Information (AMI), Adjusted Rand Index (ARI), Average Silhouette Width (ASW), and Cumulative Variance Ratio (CVR) (see ‘Materials and methods’ section for details on each metric). These metrics approach a value of 1.0 when cells segregate into well-resolved and homogenous groupings reflective of cell types. We then performed hierarchical clustering on the z-scores for these metrics to compare kits (Figure 3D). These analyses revealed that 10 × 3′, 10 × 5′, 10x FRP, Fluent and BD exhibit relatively high scores, associated with well-resolved cell labels in discrete clusters (Extended Data Figure 6l, m), in contrast to Honeycomb, Parse, Scale and Scipio (Supplementary Table 18).

Next, we sought to determine the impact of kit-specific cell partitioning on differential gene expression among cell types. We reasoned that kits capable of generating discrete and homogeneous cell groupings would result in more sensitive detection of differentially expressed genes (DEGs). Across all cell types, 10× FRP and 10 × 3′ yielded the highest number of DEGs, while Honeycomb and Scipio exhibited the lowest number of DEGs (Figure 3E; Supplementary Table 19). Interestingly, Fluent exhibited high variation across cell types, showing abundant DEGs for monocytes and dendritic cells but few for all other cell types.

## Discussion

**Figure 4.**
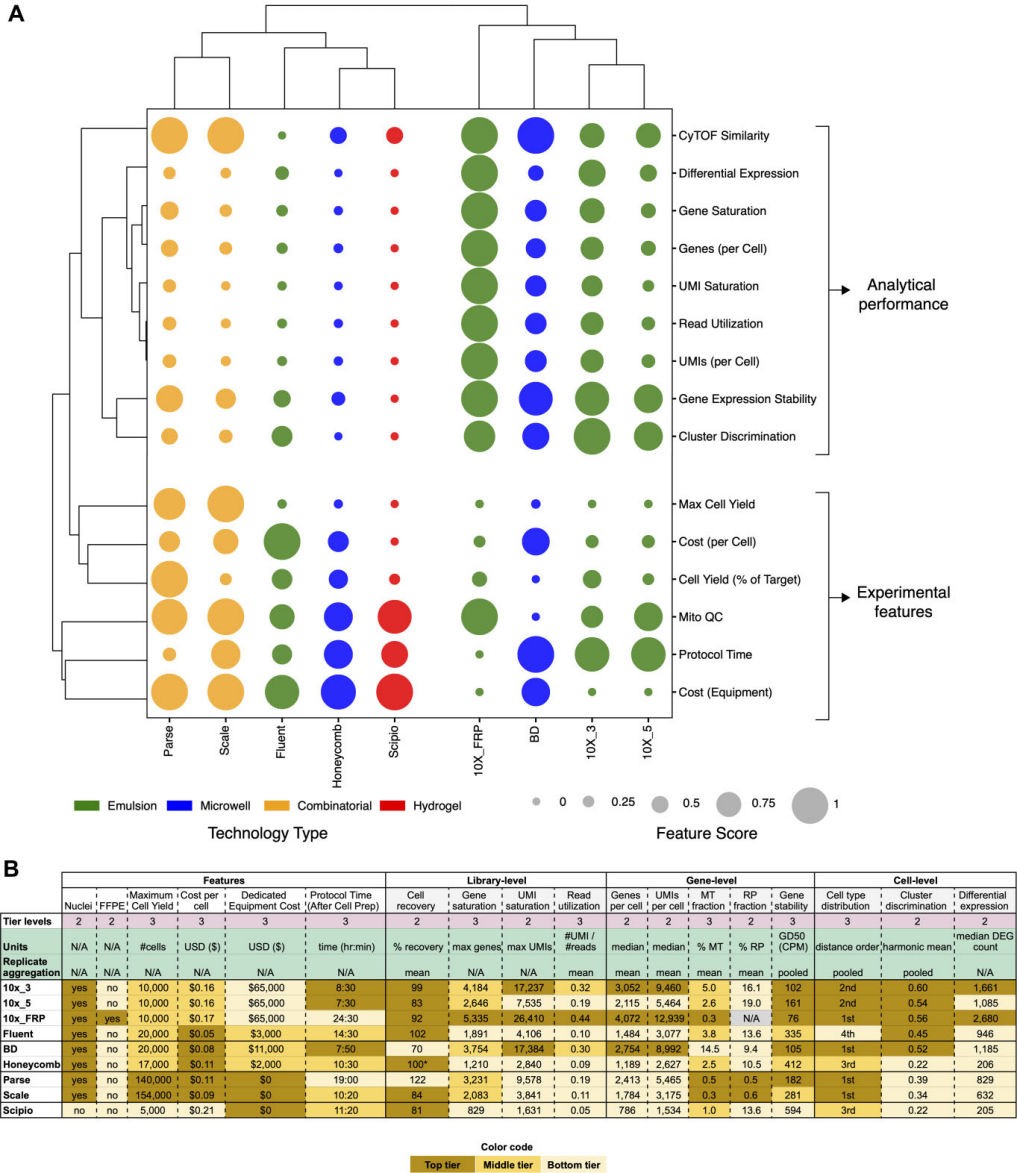
Summary of key features and performance results. (**A**) scRNA-seq kit feature scores ordered by hierarchical clustering dendrograms. The relative feature scores are derived from continuous metric values and normalized to between 0 (non-optimal; smaller circle) and 1 (optimal; larger circle). The optimal feature score is inversely proportional to the metric value for the following: Protocol Time, Cost (Equipment), Cost (per Cell), Mito QC, CyTOF Similarity (cophenetic distance score) and Gene Expression Stability (Gene Dropout GD50). (**B**) Table of metric values colored by rankings and broken into groups. Details about the tiering, units, and aggregation method are provided at the top. The methods used to define tiering can be found in the Materials & Methods section under *Tiering System*. Asterisk (*) indicates that observed cell recovery results should be considered with caution since the cell calling software returns the user-defined number of targeted cells rather than empirically estimating valid CBs.

Recent advancements in scRNAseq technologies have enabled researchers to choose between a diverse set of technologies to meet their specific investigative needs. However, with this diversification, choosing between available technologies can pose a challenge. Using a PBMC sample from a single donor blood draw, we performed a systematic comparison of currently available commercial kits to characterize features and performance metrics relevant to selecting an appropriate scRNA-seq approach. To facilitate the comparison of kits, we have provided a summary of relevant features in addition to library-, gene- and cell-level performance metrics (Table 1; Figure 4).

### Analytical performance

Overall, 10x FRP exhibited the highest ranked analytical performance followed by 10 × 3′, BD, Scale, 10 × 5′ / Parse (tie), Fluent, Honeycomb and Scipio. Interestingly, at the gene level, we found a significant reduction in MT and RP fractions in kits that require cell fixation and permeabilization (10× FRP, Parse and Scale). We hypothesized that cell fixation and permeabilization lead to loss of cytosolic transcripts including MT and RP transcripts, which was corroborated by an observed increase in the proportion of intronic reads likely derived from unspliced transcripts in the nucleus. Additionally, we noticed that BD yielded a distinguishingly high MT fraction. Because relatively high MT and RP fractions are commonly used to identify low-quality cells in scRNA-seq data, kits exhibiting lower MT and RP fractions were scored favorably in Figure 4. Note that all results are based on a single PBMC sample, so the observed variation in MT and RP fractions between kits is the result of technical factors (e.g. missing probes, fixation, etc.); therefore, it is important to select cell quality thresholds that account for kit-specific biases. Lastly, while most kits exhibited consistent ranked performance across individual cell-level metrics, Fluent displayed high variation. Despite exhibiting the highest deviation in cell type distribution relative to CyTOF, Fluent demonstrated discrete cell partitioning and moderate differential gene expression sensitivity.

With respect to the transcript detection method, 10x FRP is unique among the evaluated kits because it utilizes probe hybridization to RNA rather than RT. While the use of probe hybridization is associated with the superior performance demonstrated by 10× FRP, it is currently limited to the detection of target coding regions in human and mouse genomes. In addition, probe hybridization is currently incompatible with characterization of expressed genetic variation, which precludes the application of genotype-based demultiplexing tools (42,43). Lastly, the variable number of probes and target sequences for each gene may introduce bias in expression quantitation. For RT-based kits, the extent of gene body read coverage is critical to genotyping and specialized applications such as TCR/BCR repertoire analysis. While most RT-based kits exhibited biased coverage to either the 3′- or 5′-end of genes, Parse and Honeycomb demonstrated relatively uniform coverage throughout gene bodies due to the use of random hexamers during cDNA generation (Extended Data Figure 7).

### Experimental features

Another set of distinguishing features relates to sample type compatibility. Here, again, 10× FRP is distinct due to its ability to process cells, nuclei, and formalin-fixed paraffin-embedded (FFPE) tissue. Its unique ability to process FFPE tissue greatly expands the reach of scRNA-seq to access the vast catalogs of preserved clinical specimens. Relatedly, 10× FRP and the combinatorial-indexing kits, Parse and Scale, require sample fixation, which enables asynchronous sample acquisition and storage followed by batch processing. In general, batch processing of either fixed or fresh samples improves efficiency and increases scale. For example, a high-throughput variant of the 10× FRP kit can be configured with barcoded probe sets to enable simultaneous processing of up to 16 samples while still allowing the capture of up to 10 000 cells per sample. Similarly for Parse and Scale, plate-based sample barcoding and combinatorial-indexing enables batch processing of up to 96 samples. However, the labor-intensive process of split-pool barcoding requires a high number of input cells due to sample loss during successive rounds of washing and barcoding. Another multiplexing technique is cell hashing which utilizes barcoded antibodies originally developed for emulsion-based methods (44) such as 10 × 3′ and 10 × 5′ but is amenable to other scRNA-seq technology groups. In addition, cell hashing enables the detection of multiplets, which is especially useful for maximizing cell recovery yield (30) and further reducing cost per cell.

### Cost analysis

While analytical performance and experimental features define assay utility, cost is among the most salient factors in selecting a scRNA-seq kit. To compare kits using a common basis, we computed cost per cell by dividing the cost of a single reaction by the claimed maximum cell yield, excluding the cost of sequencing (Figure 4B). This analysis revealed Fluent as the most cost-efficient kit, followed by BD and Scale, Honeycomb and Parse, the 10× variants, and lastly Scipio. It is worth noting that higher-throughput kit versions along with specific experimental configurations, such as cell overloading, can further reduce cost by increasing cell recovery yield. Additionally, there are upfront equipment costs associated with each kit that contribute to expense assessment and these costs vary drastically among kits. For instance, the dedicated equipment for the 10x kits is around $65 000 while Scipio, Parse and Scale do not require specialized instrumentation (Table 1). Therefore, a holistic expense assessment must consider the costs of both procuring equipment in addition to projected kit consumption over time. When averaging upfront equipment and kit costs over expected output ranging from 10^4^ to 10^7^ cells, the Fluent kit emerges as the most cost-efficient; whereas, the 10× Genomics platform expenses generally exceed all other kits (except Scipio beyond about 1.3 million cells) (Supplementary Table 20a). Importantly, these cost estimates are based on the features of the currently evaluated kits. The use of higher throughput variants of these kits (e.g. 16-plex 10x FRP) together with other multiplexing strategies will dramatically impact the cost structure. Moreover, even though we omitted sequencing costs in our calculations, we identified substantial variation in aspects of read utilization among kits, which can impact cost per cell. For instance, about 90% of reads from the 10× FRP kit were usable (mapped and CB-tagged); whereas, Fluent utilizes less than 40% of reads. This inefficiency more than doubles the sequencing cost per cell to achieve the same number of usable reads, assuming a constant saturation rate. Library complexity is another factor that impacts read utilization, as only unique molecules are counted (Extended Data Figure 4c). Taken together, read utilization is an important metric that concisely describes the efficiency of converting sequencing reads into usable counts, which determines sensitivity and cost-effectiveness.

### Protocol duration

We estimated protocol duration based on published user guides, starting from either fresh or fixed cells and ending with completed libraries, which revealed that current commercially-available scRNA-seq kits require at least about 8 h (10 × 3′, 10 × 5′ and BD) and as long as 24.5 h (10× FRP; accounting for an overnight incubation step). Analysis of the cell processing rate (maximum cell yield over protocol duration) indicates that the 10x FRP protocol requires about 36-fold more time than Scale, the most time-efficient kit, to process an equivalent number of cells (Supplementary Table 20b). Depending upon anticipated experimental throughput, factors such as upfront equipment cost, kit cost and protocol time are important variables in selecting an appropriate scRNA-seq method.

### Relationships among kits, experimental features and analytical performance

The insights from these analyses are aggregated in Figure 4A. Hierarchical clustering along both kit and metric axes reveals two cohorts characterized by high- versus low- analytical performance. The former cohort is composed of 10x FRP, 10 × 3′, 10 × 5′ and BD, representing the most sensitive and accurate kits. Conversely, the constituent kits of the latter cohort are associated by kit features such as cell yield, cost and protocol length. Importantly, kits in this low-analytical performance cohort have generally lower dedicated equipment costs and lower costs per cell (excluding Scipio, which has limited economies of scale due to its low-throughout design targeting 5000 cells or less). In addition, the combinatorial-indexing kits from Parse and Scale form a sub-cluster distinguished by markedly higher maximum cell yield compared to all other kits. The relationships among kits, features and performance metrics presented in Figure 4 provide a concise unified reference for selecting an appropriate scRNA-seq kit.

### Limitations

This study provides a comprehensive and systematic evaluation of commercially available scRNA-seq kits and sheds light on the various strengths and weaknesses of each kit with respect to time, cost, functionality and performance, which should facilitate selection of a suitable scRNA-seq approach. However, there are important limitations to the scope of work and interpretation of results. First, while insights derived from PBMCs provide a useful perspective, other sample types such as nuclei, fixed tissue and whole blood may reveal an alternative assessment of relative kit performance. Second, we observed an unusually high fraction of monocytes in our PBMC sample compared to the typical distribution found in healthy donors (12); however, this over-representation of monocytes was corroborated by CyTOF and was relatively consistent across kits. Third, in order to focus resources on evaluating a broad variety of kits, we did not pursue multiplet rate analysis, which would have required additional ‘barnyard’ experiments (e.g. mixed human and mouse cells). While we do not provide empirical multiplet rates, simulation-based estimates are provided (Supplementary Table 21). Lastly, we observed substantial cell recovery irregularities using the data processing pipelines provided by Fluent and Honeycomb, which may impact performance assessments by failing to exclude invalid CBs and ambient RNA. Given the ever-expanding set of diverse scRNA-seq methods, further research into technology-agnostic cell calling algorithms would be an important contribution to improving accuracy and consistency in the field.

### Summary

In this study, we benchmarked commercially-available scRNA-seq kits to enable researchers to select the appropriate technology for their experimental needs. In addition, by identifying the strengths and weaknesses of each methodology, we expect that this knowledge will help advance scRNA-seq technology development. Beyond the current set of analyses, this work provides a rich dataset consisting of 169, 262 called cells across nine kits with 119, 428 cell type annotations. Such a dataset can be used to train annotation models or to benchmark algorithms that explicitly account for technical variability among scRNA-seq approaches. Taken together, we have established a comprehensive experimental and analytical framework for comparing scRNA-seq technologies. The insights and underlying data generated from this work have the potential to facilitate substantial progress in the field of single-cell biology.

## Supplementary Material

gkae1186_Supplemental_Files

## Data Availability

Sequencing data were deposited to NCBI Sequence Read Archive (SRA) under the BioProject accession PRJNA1106903. Single-cell gene expression data were deposited to CZ CELLxGENE under the Collection accession 398e34a9-8736-4b27-a9a7-31a47a67f446.

## Code availability

The code used to analyze data from this study has been deposited in the GitHub repository: https://github.com/danledinh/scRNAseq-repo (Zenodo DOI: 10.5281/zenodo.13910293).
