# Supplementary material for: A comprehensive analysis framework for evaluating commercial single-cell RNA sequencing technologies
Source: Nucleic Acids Res. 2024 Dec 16;53(2):gkae1186. doi: 10.1093/nar/gkae1186 (PMC11754665; doi:10.1093/nar/gkae1186)
Supplement: gkae1186_Supplemental_Files [file gkae1186_supplemental_files.zip › EDF_captioned.pdf]

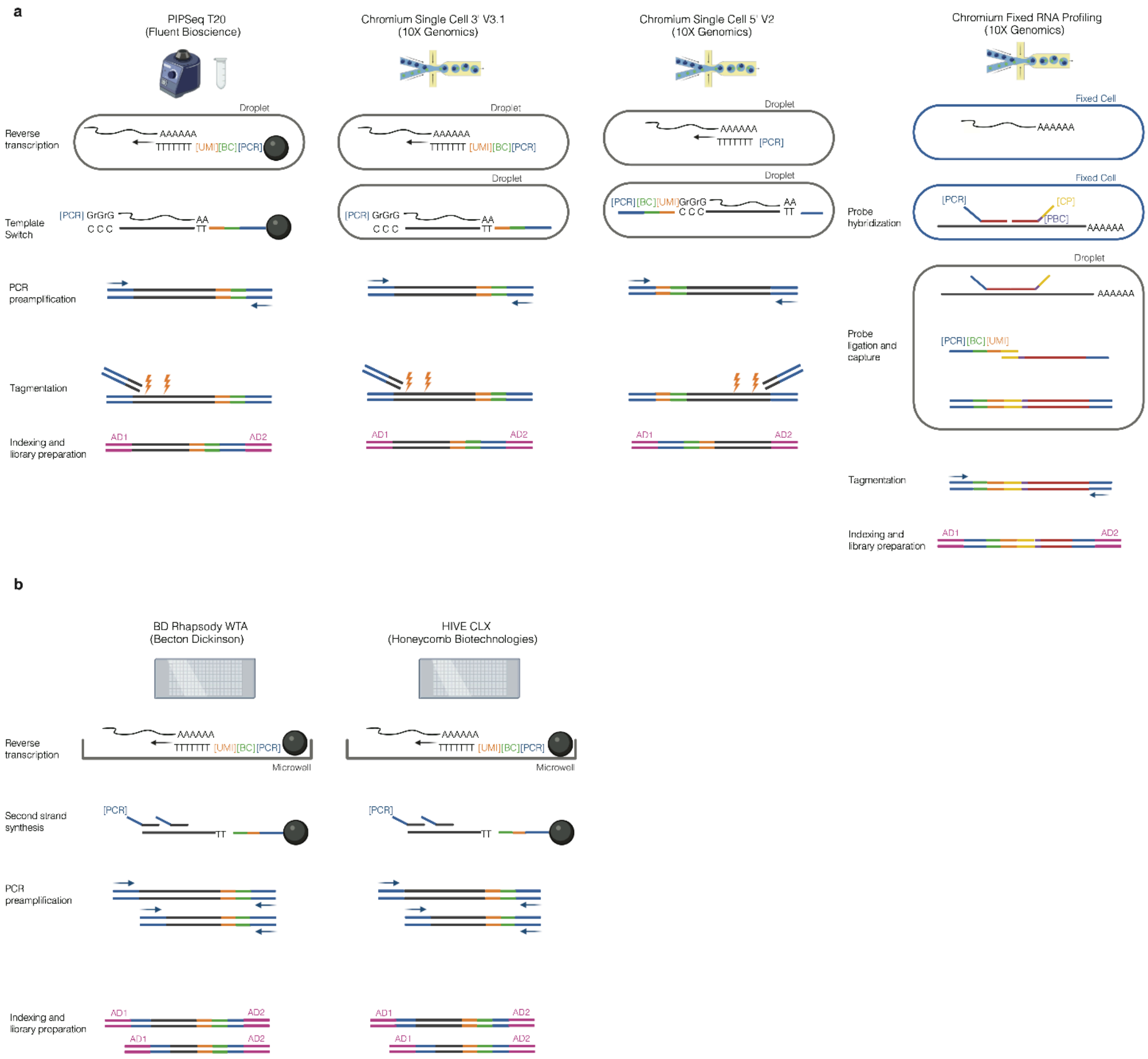

a
PIPSeq T20
(Fluent Bioscience)
Chromium Single Cell 3' V3.1
(10X Genomics)
Chromium Single Cell 5' V2
(10X Genomics)
Chromium Fixed RNA Profiling
(10X Genomics)
Droplet
Fixed Cell
Reverse transcription
AAAAAA
TTTTTTT [UMI][BC][PCR]
TTTTTTT [PCR]
Template Switch
[PCR] GrGrG
C C C
AA
TT
[PCR] [BC] [UMI]GrGrG
Probe hybridization
[PCR]
[CP]
[PBC]
PCR preamplification
Probe ligation and capture
[PCR] [BC] [UMI]
Tagmentation
Indexing and library preparation
AD1
AD2
b
BD Rhapsody WTA
(Becton Dickinson)
HIVE CLX
(Honeycomb Biotechnologies)
Reverse transcription
AAAAAA
TTTTTTT [UMI][BC][PCR]
Microwell
Second strand synthesis
[PCR]
TT
PCR preamplification
Indexing and library preparation
AD1
AD2

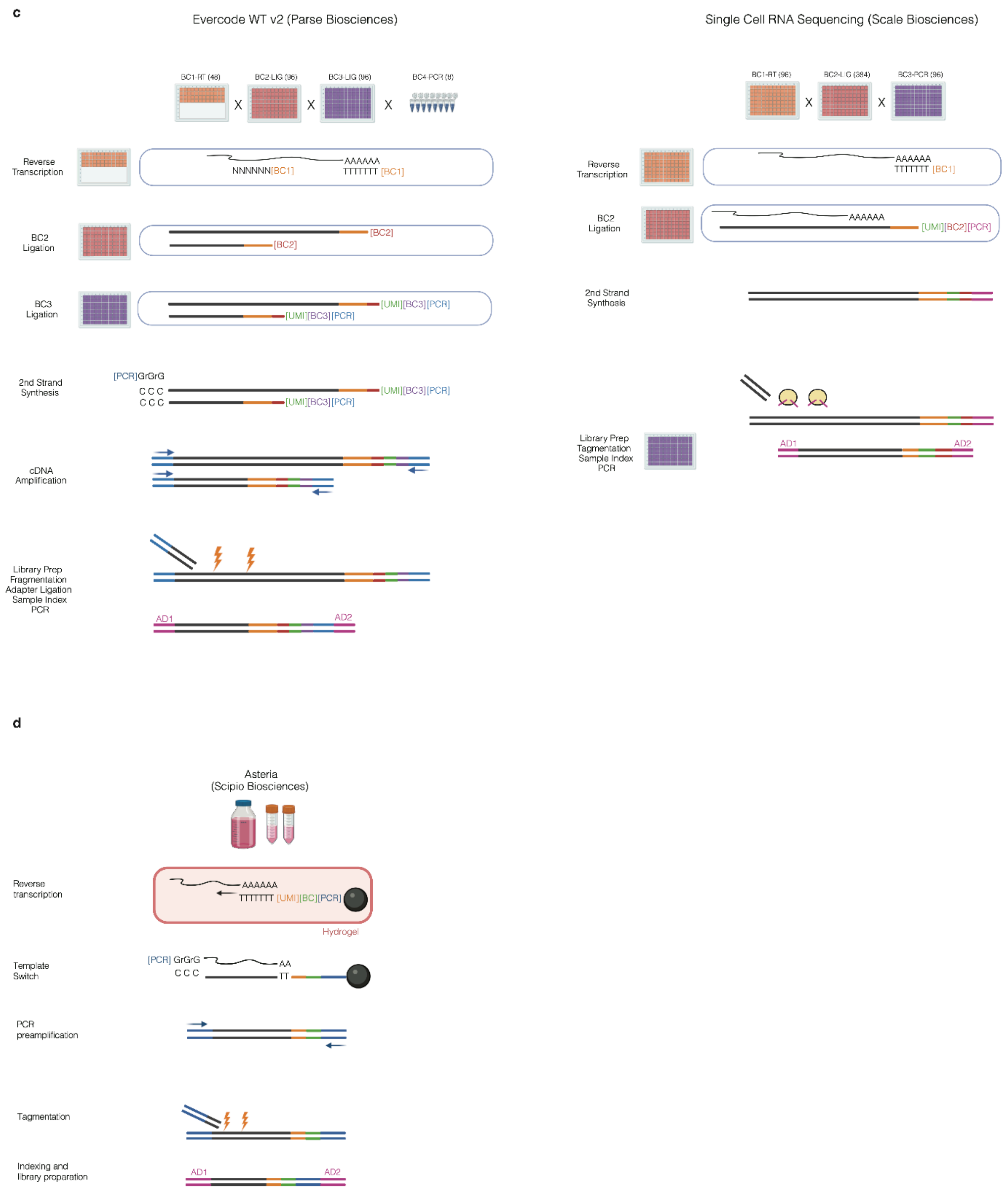

Extended Data Figure 1. Schematics of the single-cell RNA sequencing protocols tested. Protocols were classified into four categories according to the method of cell isolation: **a)** Emulsion-based protocol, **b)** Microwell-based protocols, **c)** Combinatorial-indexing protocols, and **d)** Hydrogel-based protocol. BC: barcode; UMI: unique molecular identifier; AD: adapter; CP: capture probe; PCR: polymerase chain reaction handle; PBC: probe barcode.

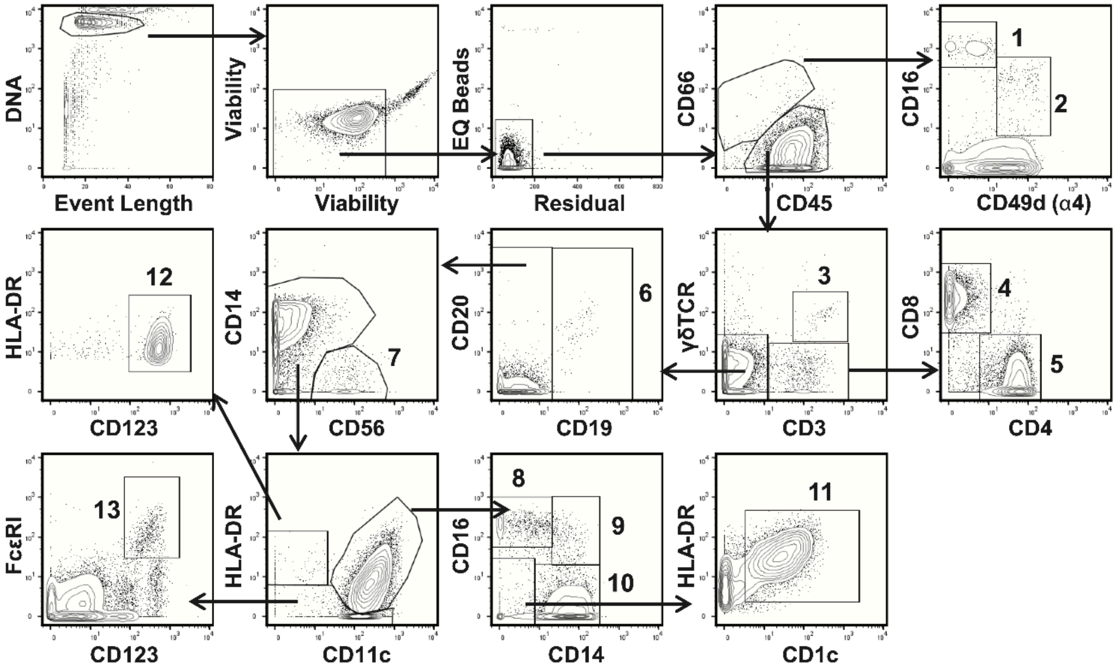

1. Neutrophil
2. Eosinophil
3. γδ T cells
4. CD8 T cells
5. CD4 T cells
6. CD19 B cells
7. NK cells
8. CD16 Mono
9. Intermediate Mono
10. CD14 Mono
11. mDCs
12. pDCs
13. Basophils

Extended Data Figure 2. CyTOF gating strategy. Gating strategy used to calculate the PBMC subpopulation frequencies. See **Supplementary Table 20a,b** for details regarding antibodies and markers used to identify the subpopulations.

a

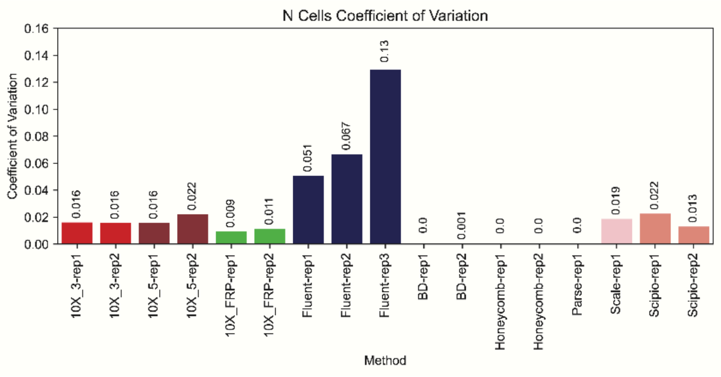
N Cells Coefficient of Variation
Coefficient of Variation
0.16
0.14
0.12
0.10
0.08
0.06
0.04
0.02
0.00
0.016
0.016
0.016
0.022
0.009
0.011
0.051
0.067
0.13
0.0
0.001
0.0
0.0
0.0
0.019
0.022
0.013
10X_3-rep1
10X_3-rep2
10X_5-rep1
10X_5-rep2
10X_FRP-rep1
10X_FRP-rep2
Fluent-rep1
Fluent-rep2
Fluent-rep3
BD-rep1
BD-rep2
Honeycomb-rep1
Honeycomb-rep2
Parse-rep1
Scale-rep1
Scipio-rep1
Scipio-rep2
Method

b

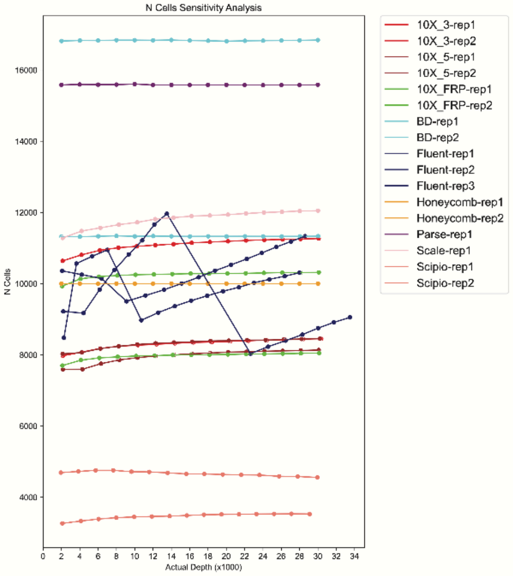
N Cells Sensitivity Analysis
N Cells
16000
14000
12000
10000
8000
6000
4000
0 2 4 6 8 10 12 14 16 18 20 22 24 26 28 30 32 34
Actual Depth (x1000)
10X_3-rep1
10X_3-rep2
10X_5-rep1
10X_5-rep2
10X_FRP-rep1
10X_FRP-rep2
BD-rep1
BD-rep2
Fluent-rep1
Fluent-rep2
Fluent-rep3
Honeycomb-rep1
Honeycomb-rep2
Parse-rep1
Scale-rep1
Scipio-rep1
Scipio-rep2

c

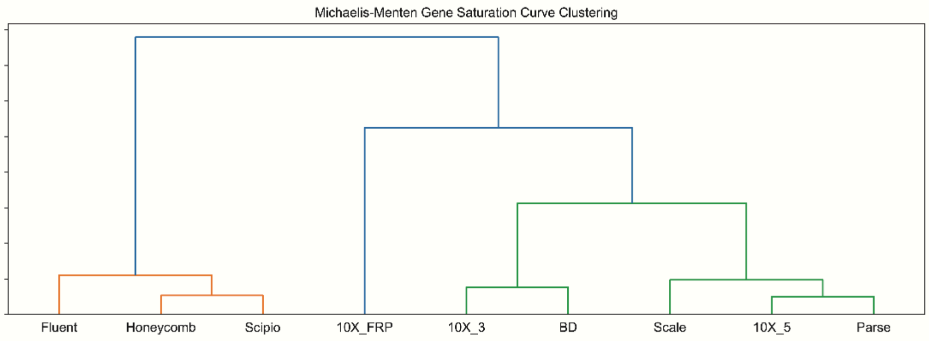
Michaelis-Menten Gene Saturation Curve Clustering
Fluent
Honeycomb
Scipio
10X_FRP
10X_3
BD
Scale
10X_5
Parse

d

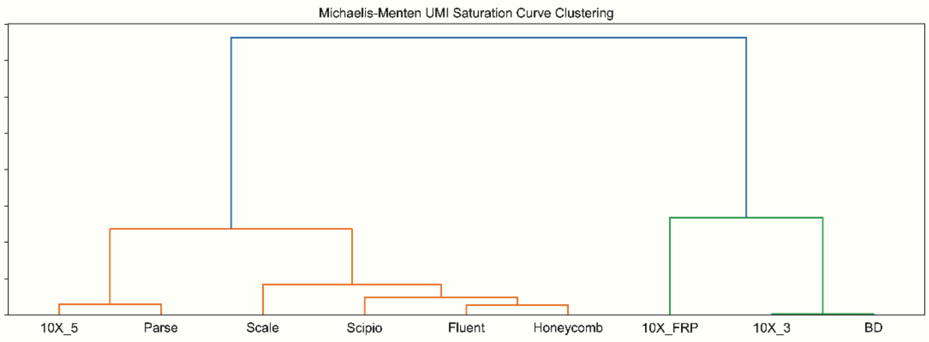
Michaelis-Menten UMI Saturation Curve Clustering
10X_5
Parse
Scale
Scipio
Fluent
Honeycomb
10X_FRP
10X_3
BD

Extended Data Figure 3. Downsampling sensitivity analysis. a) Coefficient of variation for number of cells detected across all downsampled depths (30,000 to 2,000 in steps of 2,000 reads per cell). **b)** Number of cells recovered at each sampling depth (according to cell calling algorithms accompanying each kit). **c)** Hierarchical clustering dendrogram for the gene saturation fitted curve. **d)** Hierarchical clustering dendrogram for the UMI saturation fitted curve. Hierarchical clustering performed on 34 equidistant fitted values along the curve of each kit.

a

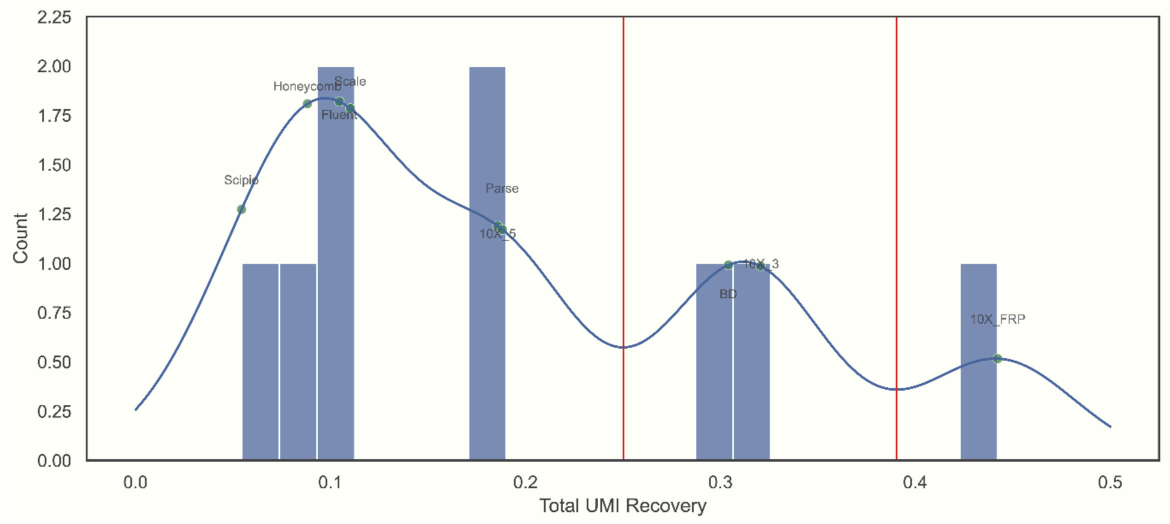

Count
Total UMI Recovery
Honeycomb
Scale
Fluent
Scipio
Parse
10X_5
10X_3
BD
10X_FRP

b

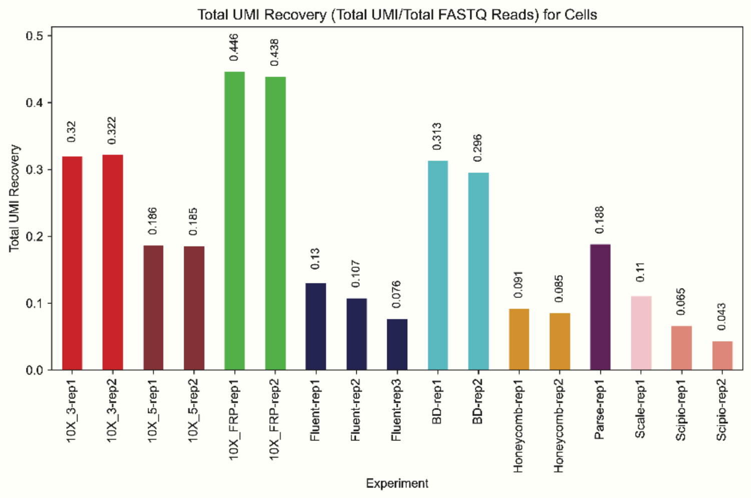

Total UMI Recovery (Total UMI/Total FASTQ Reads) for Cells
Total UMI Recovery
Experiment
0.32
0.322
0.186
0.185
0.446
0.438
0.13
0.107
0.076
0.313
0.296
0.091
0.085
0.188
0.11
0.065
0.043
10X_3-rep1
10X_3-rep2
10X_5-rep1
10X_5-rep2
10X_FRP-rep1
10X_FRP-rep2
Fluent-rep1
Fluent-rep2
Fluent-rep3
BD-rep1
BD-rep2
Honeycomb-rep1
Honeycomb-rep2
Parse-rep1
Scale-rep1
Scipio-rep1
Scipio-rep2

c

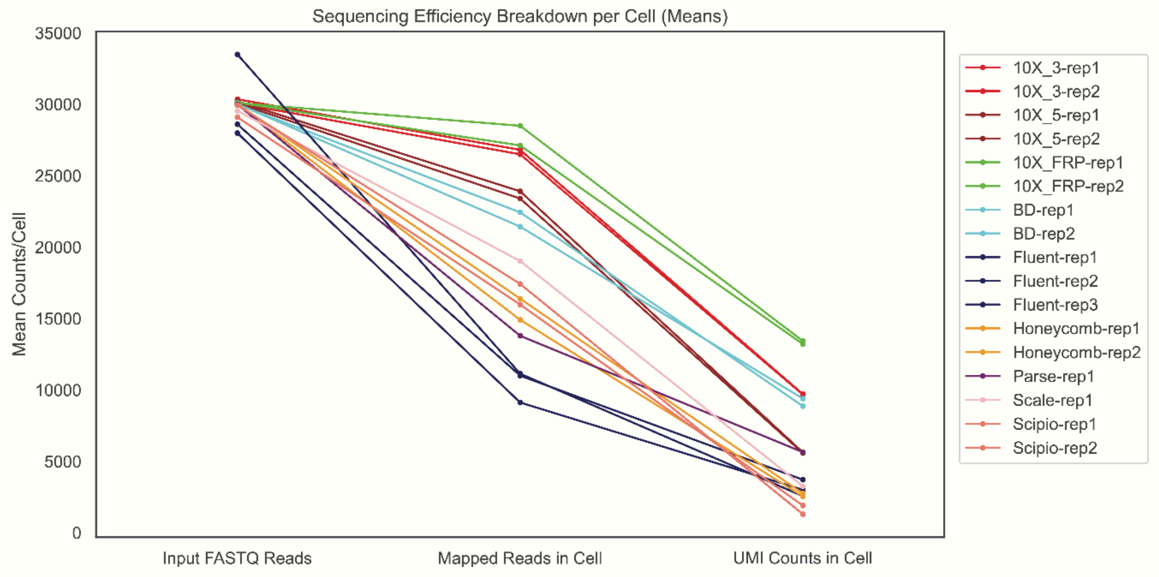

Sequencing Efficiency Breakdown per Cell (Means)
Mean Counts/Cell
Input FASTQ Reads
Mapped Reads in Cell
UMI Counts in Cell
10X_3-rep1
10X_3-rep2
10X_5-rep1
10X_5-rep2
10X_FRP-rep1
10X_FRP-rep2
BD-rep1
BD-rep2
Fluent-rep1
Fluent-rep2
Fluent-rep3
Honeycomb-rep1
Honeycomb-rep2
Parse-rep1
Scale-rep1
Scipio-rep1
Scipio-rep2

Extended Data Figure 4. UMI recovery and read utilization a) Histogram of UMI recovery in cells (total UMIs / total input FASTQ) to identify natural breaks. **b)** UMI recovery in cells. **c)** Components of read usage per cell: mean number of input reads per cell (left), average number of mapped reads per cell (middle), and average number of recovered UMIs per cell (right).

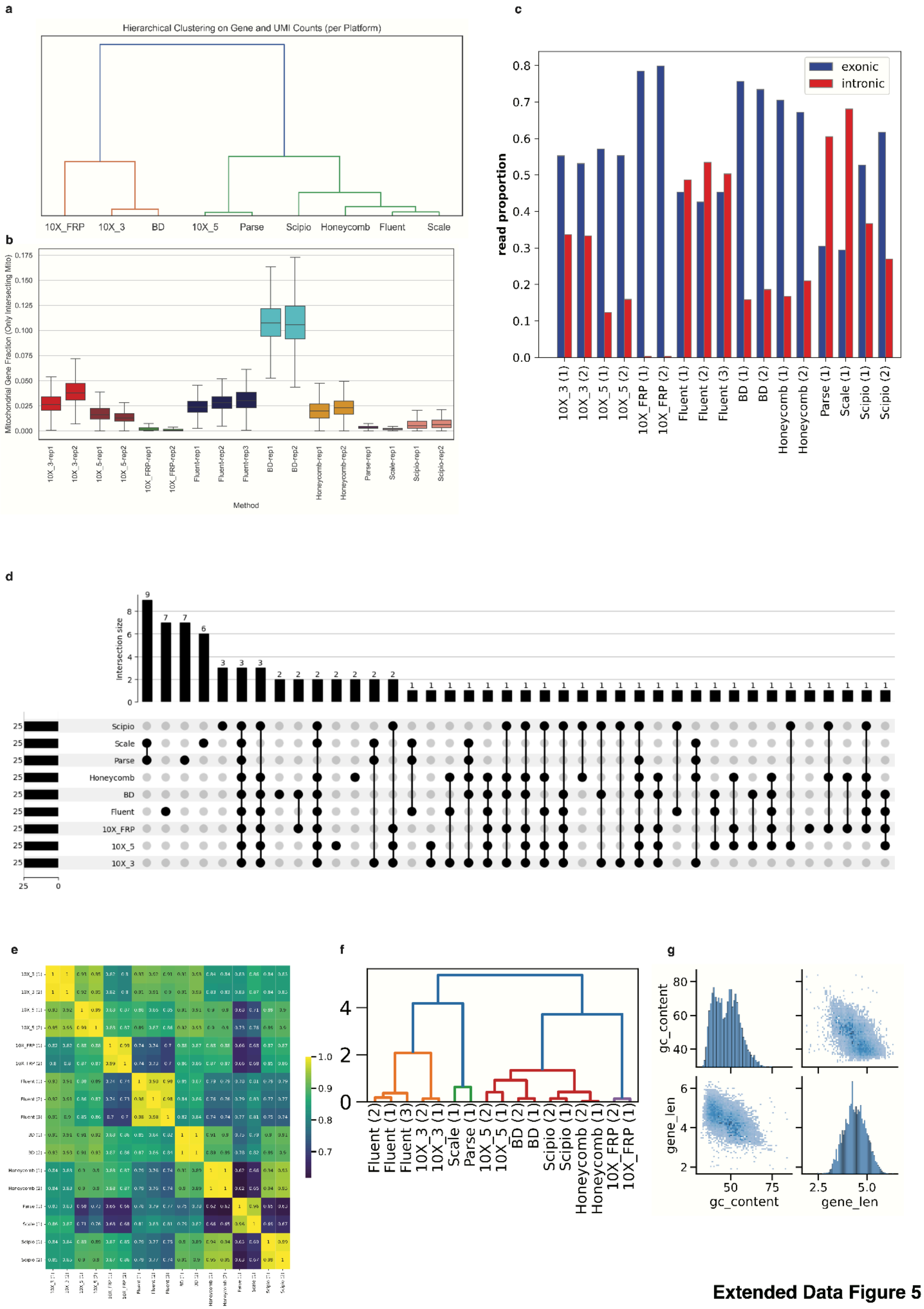

Extended Data Figure 5

h

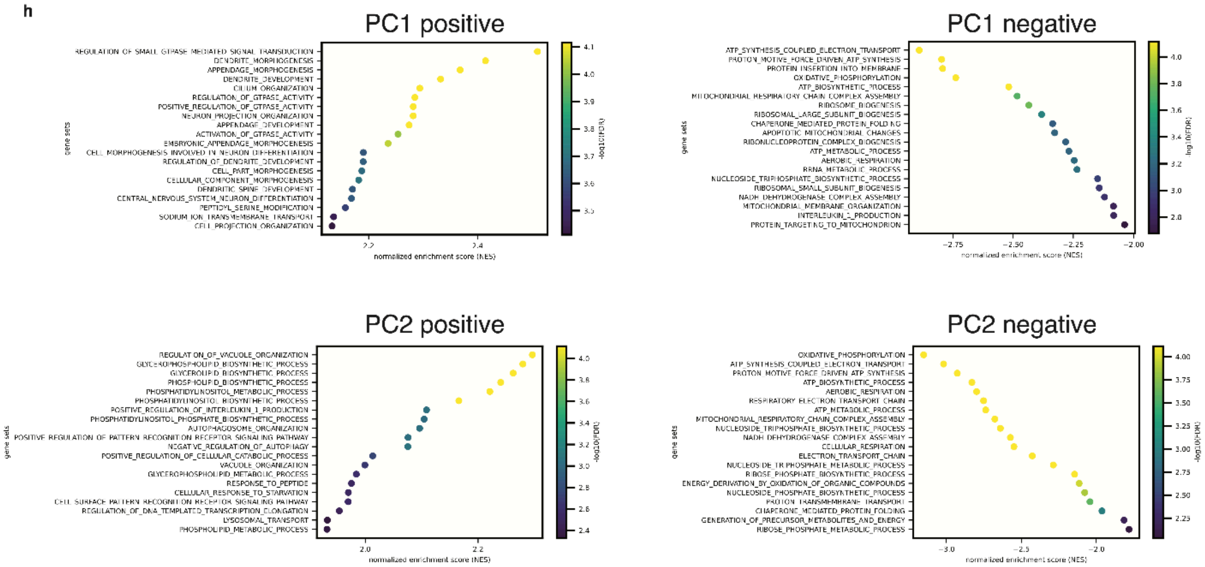

i

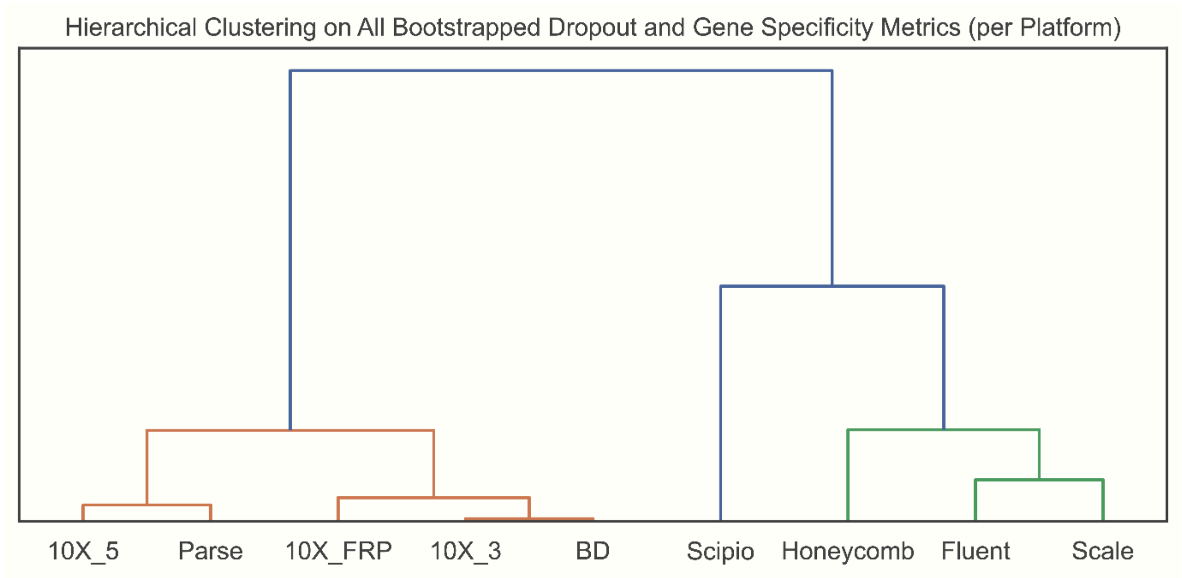

Extended Data Figure 5. Gene composition. a) Hierarchical clustering dendrogram for median gene and UMI counts of the filtered and processed 30K read depth subsample data. **b)** Fraction of MT gene counts with respect to genes common to all kits. **c)** Fraction of reads mapping to exonic (left) and intronic (right) regions. Reads must align completely within either an exon or intron. **d)** UpSet plot showing the intersections of top-25 highly-expressed genes among kits. **e)** Spearman correlations of pseudobulk gene expression profiles. **f)** Hierarchical clustering dendrogram of PC1 and PC2 embeddings. **g)** Collinearity analysis of gene length and gene GC-content. **h)** Gene set enrichment analysis of PC1 and PC2 loadings. **i)** Hierarchical clustering dendrogram of mean GD50 and mean peak gene specificity scores across 500 bootstrapped samples.

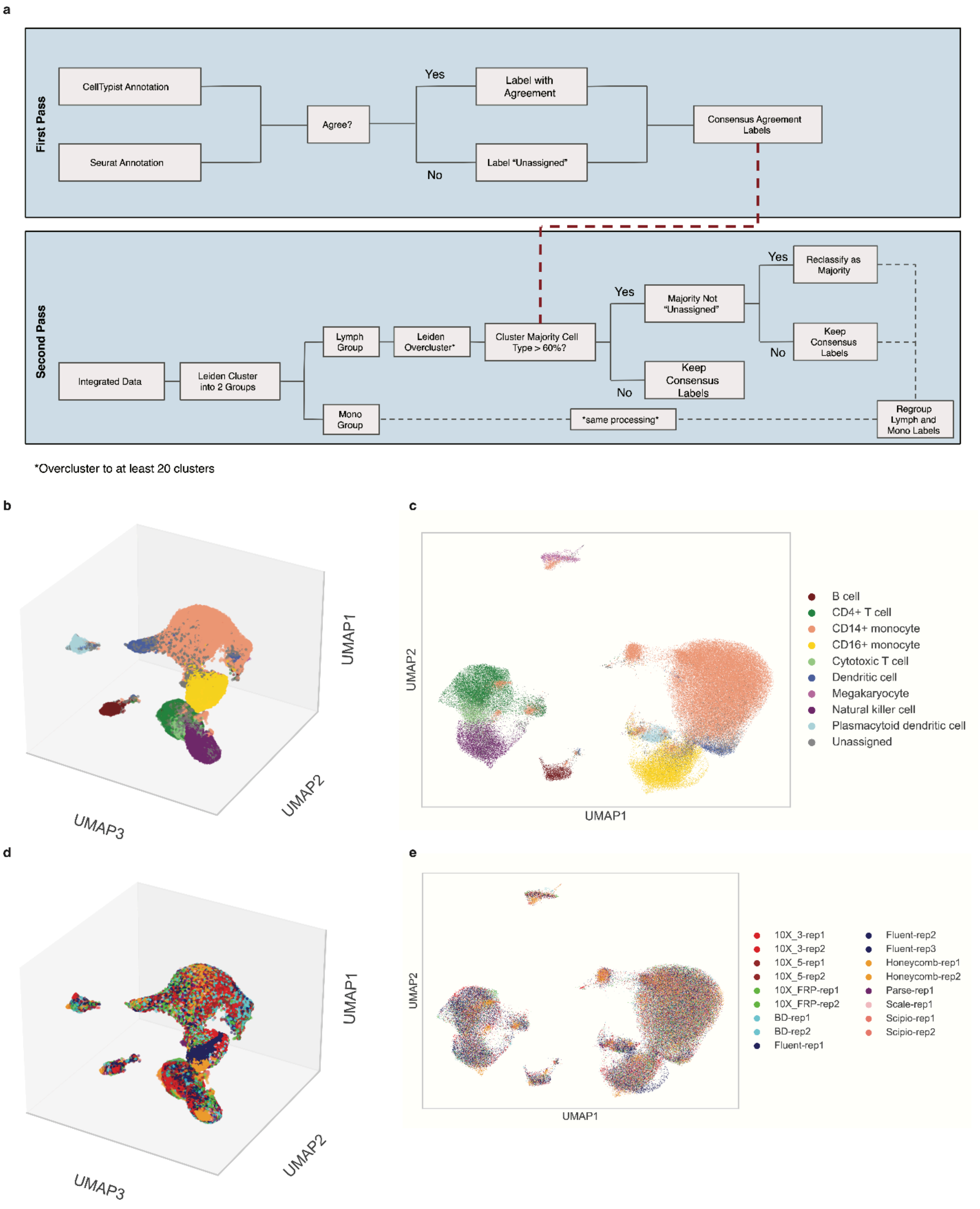
a
First Pass
CellTypist Annotation
Seurat Annotation
Agree?
Yes
No
Label with Agreement
Label "Unassigned"
Consensus Agreement Labels
Second Pass
Integrated Data
Leiden Cluster into 2 Groups
Lymph Group
Mono Group
Leiden Overcluster*
Cluster Majority Cell Type > 60%?
Yes
No
Majority Not "Unassigned"
Keep Consensus Labels
Yes
No
Reclassify as Majority
Keep Consensus Labels
same processing
Regroup Lymph and Mono Labels
*Overcluster to at least 20 clusters
b
UMAP1
UMAP2
UMAP3
c
UMAP2
UMAP1
B cell
CD4+ T cell
CD14+ monocyte
CD16+ monocyte
Cytotoxic T cell
Dendritic cell
Megakaryocyte
Natural killer cell
Plasmacytoid dendritic cell
Unassigned
d
UMAP1
UMAP2
UMAP3
e
UMAP2
UMAP1
10X_3-rep1
10X_3-rep2
10X_5-rep1
10X_5-rep2
10X_FRP-rep1
10X_FRP-rep2
BD-rep1
BD-rep2
Fluent-rep1
Fluent-rep2
Fluent-rep3
Honeycomb-rep1
Honeycomb-rep2
Parse-rep1
Scale-rep1
Scipio-rep1
Scipio-rep2

f

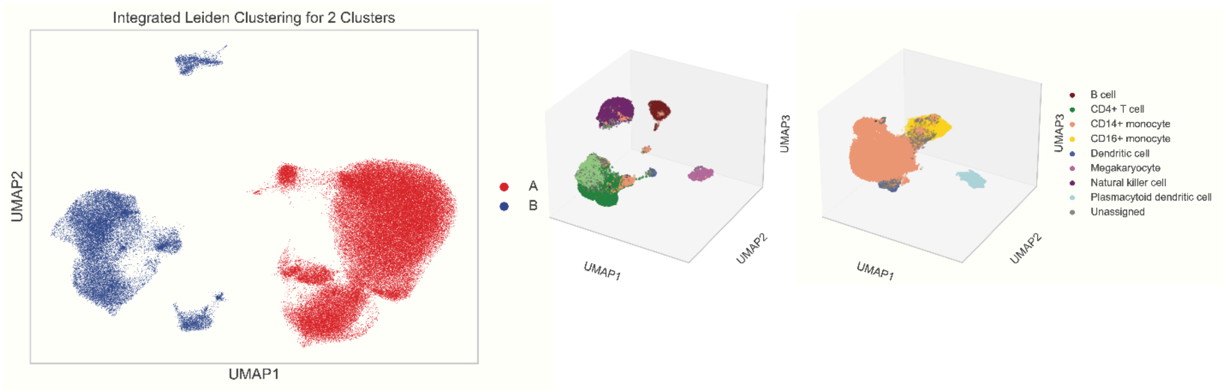
Integrated Leiden Clustering for 2 Clusters
UMAP2
UMAP1
A
B
UMAP1
UMAP2
UMAP3
UMAP1
UMAP2
UMAP3
B cell
CD4+ T cell
CD14+ monocyte
CD16+ monocyte
Dendritic cell
Megakaryocyte
Natural killer cell
Plasmacytoid dendritic cell
Unassigned

g

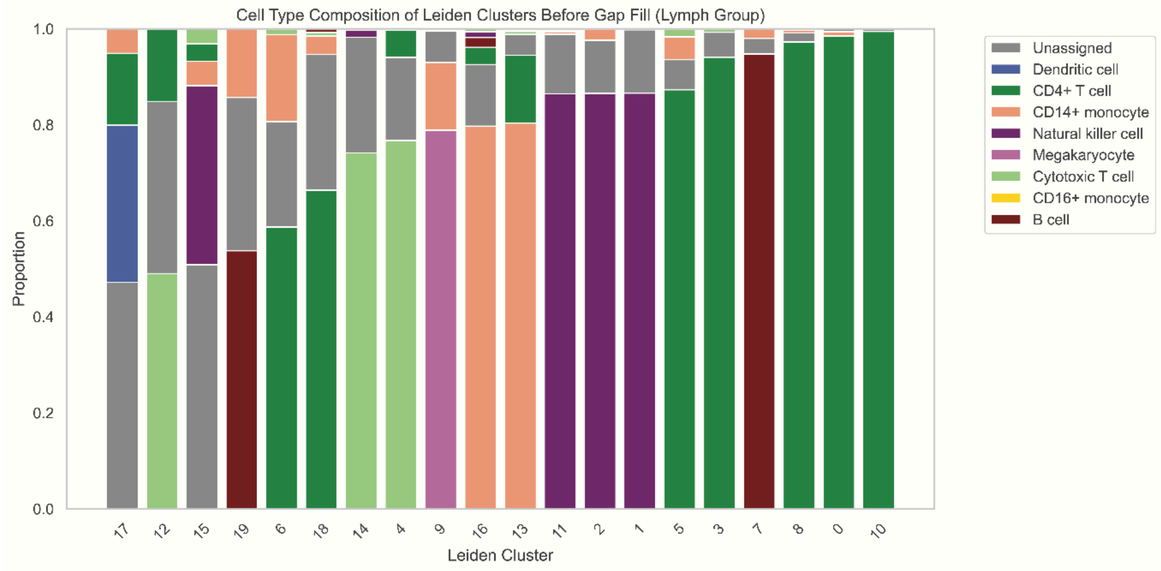
Cell Type Composition of Leiden Clusters Before Gap Fill (Lymph Group)
Proportion
0.0
0.2
0.4
0.6
0.8
1.0
17
12
15
19
6
18
14
4
9
16
13
11
2
1
5
3
7
8
0
10
Leiden Cluster
Unassigned
Dendritic cell
CD4+ T cell
CD14+ monocyte
Natural killer cell
Megakaryocyte
Cytotoxic T cell
CD16+ monocyte
B cell

h

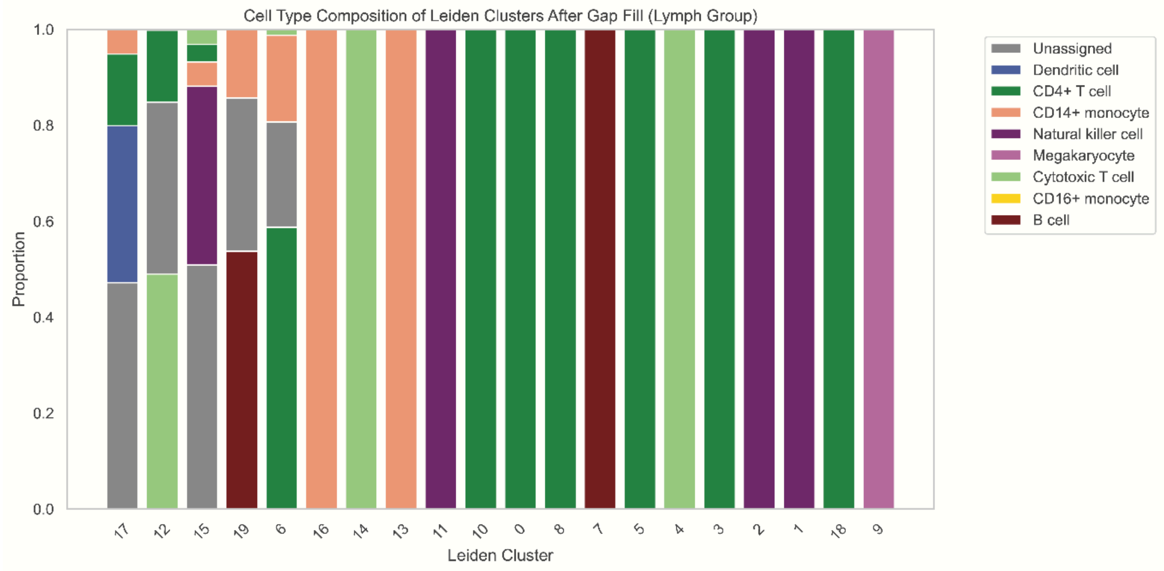
Cell Type Composition of Leiden Clusters After Gap Fill (Lymph Group)
Proportion
0.0
0.2
0.4
0.6
0.8
1.0
17
12
15
19
6
16
14
13
11
10
0
8
7
5
4
3
2
1
18
9
Leiden Cluster
Unassigned
Dendritic cell
CD4+ T cell
CD14+ monocyte
Natural killer cell
Megakaryocyte
Cytotoxic T cell
CD16+ monocyte
B cell

i

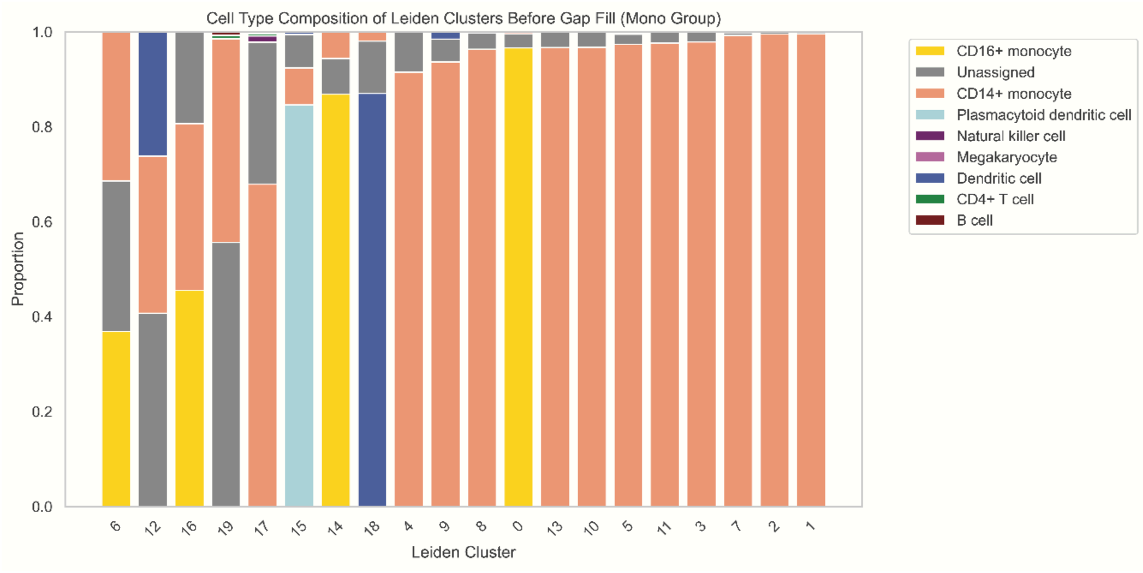

j

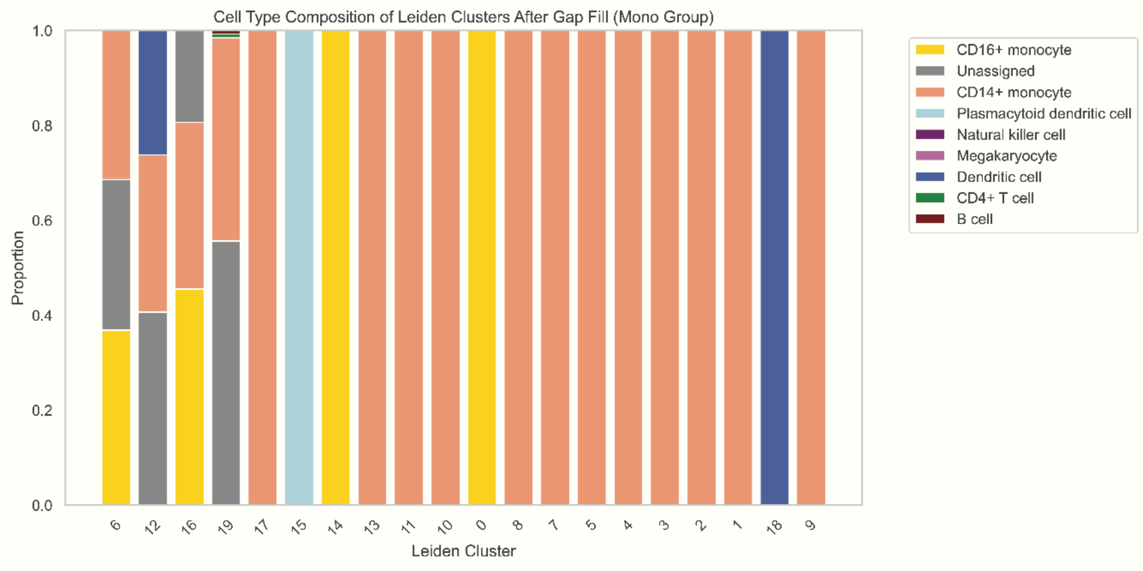

k

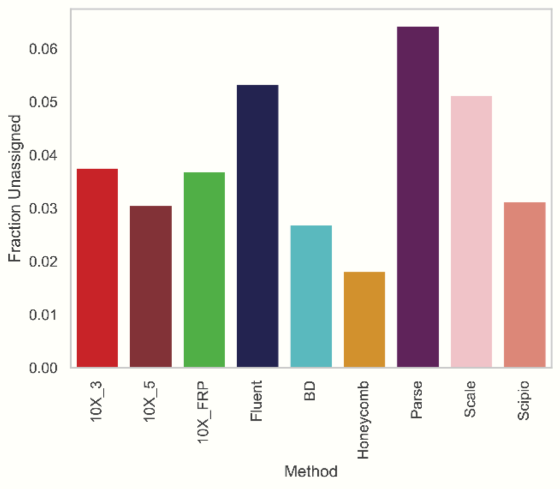

Extended Data Figure 6

I

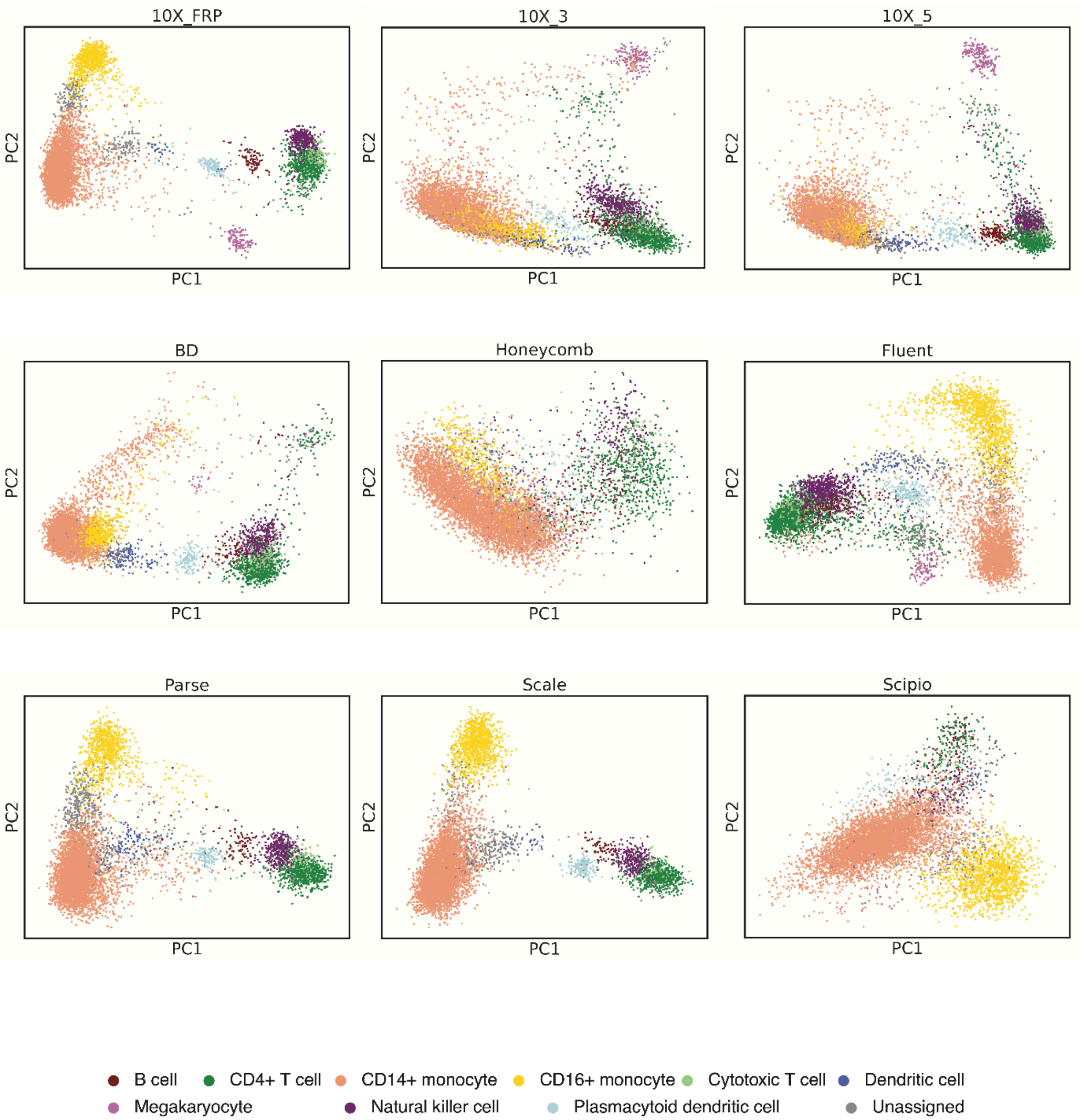
10X_FRP
10X_3
10X_5
BD
Honeycomb
Fluent
Parse
Scale
Scipio
PC1
PC2
B cell
CD4+ T cell
CD14+ monocyte
CD16+ monocyte
Cytotoxic T cell
Dendritic cell
Megakaryocyte
Natural killer cell
Plasmacytoid dendritic cell
Unassigned

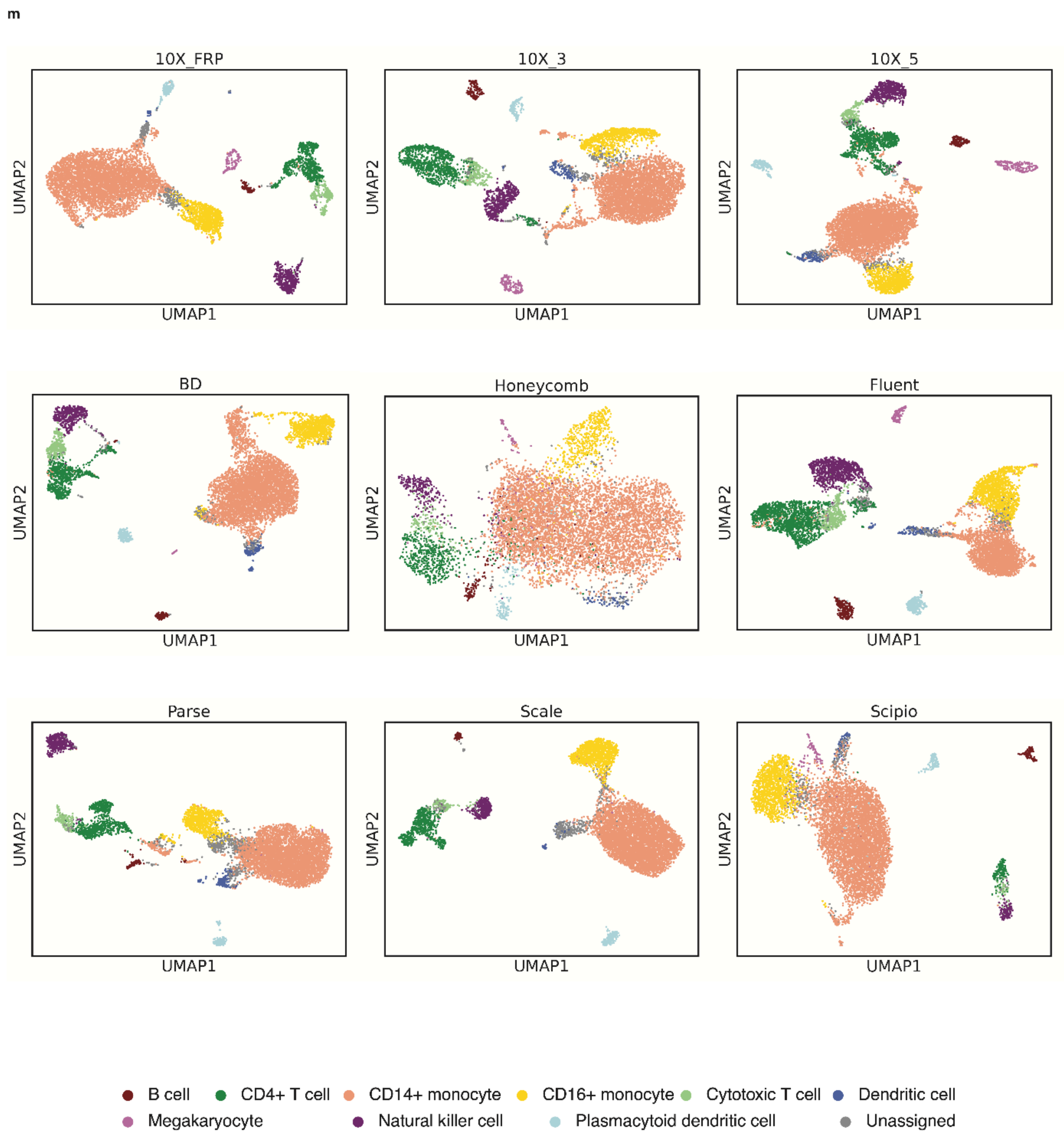

Extended Data Figure 6. Cell annotation. a) Schematic of two-pass cell annotation strategy. **b-c)** 3D and 2D UMAPs of cells colored by first-pass cell annotation results. **d-e)** 3D and 2D UMAPs of cells colored by kit after *Harmony* integration showing mixing of kits across cell types. **f)** Integrated cell population divided into lymphocyte-like cells in blue (left, group B) and monocyte-like cells in red (right, group A). Two-pass cell annotation results for the lymphocyte-like group (middle) and monocyte-like group (right). **g-h)** For lymphocyte-like population, Leiden cluster cell type composition after first- and second-pass annotation. **i-j)** As above, for the monocyte-like population. **k)** Fraction of unassigned cells remaining after annotation. **l)** 2D PCAs of each kit (independently processed and visualized) colored by the

two-pass cell annotation results. **m)** 2D UMAPs of each kit (independently processed and visualized) colored by the two-pass cell annotation results.

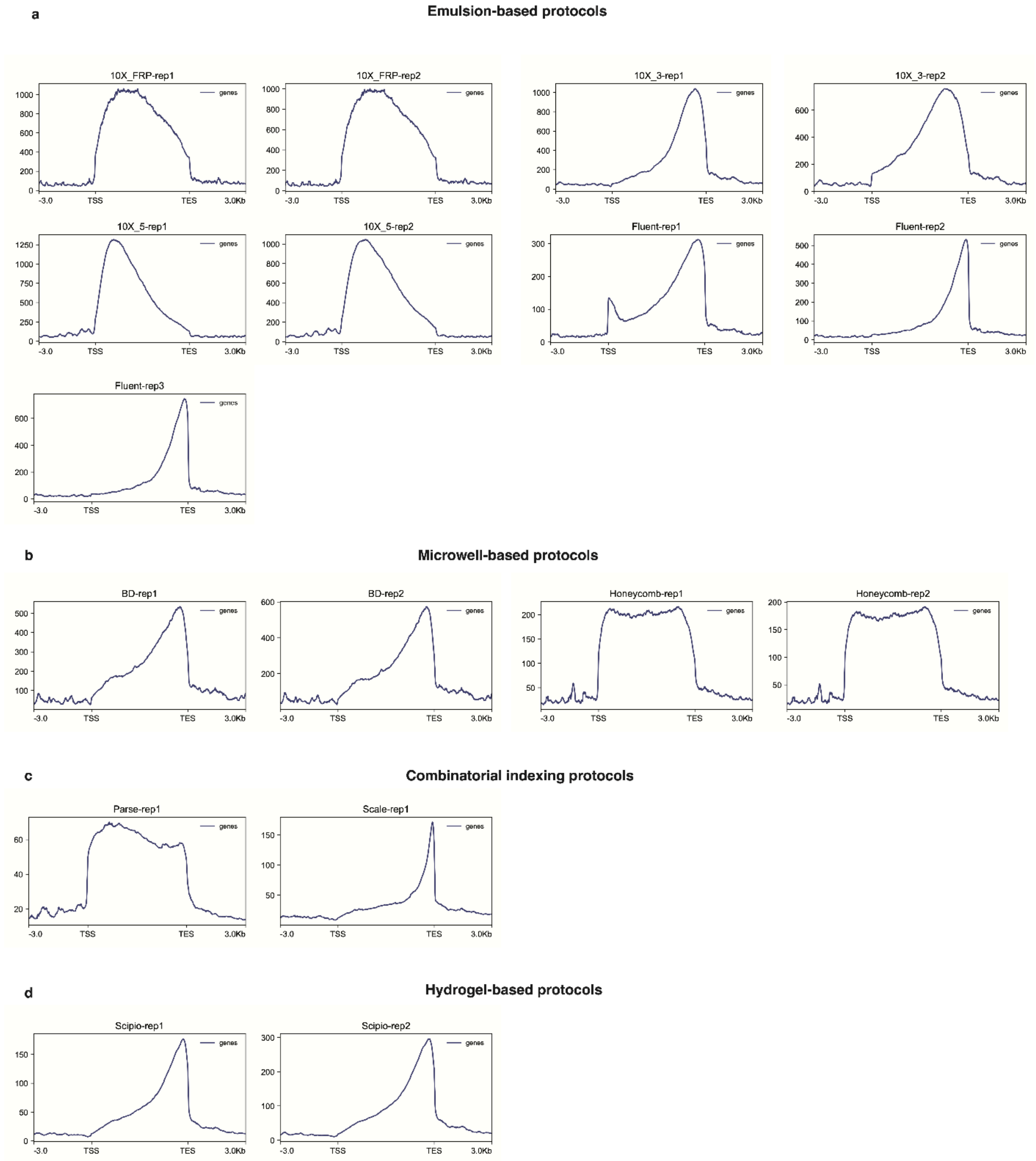

Extended Data Figure 7. Metagene coverage. Density of reads found in cells mapping to exonic regions across gene bodies. Coverage plots are organized according to technology groups: **a)** Emulsion-based protocols, **b)** Microwell-based protocols, **c)** Combinatorial-indexing protocols, and **d)** Hydrogel-based protocols.
